# Analysis of recurrent events in cluster randomised trials: The PLEASANT trial case study

**DOI:** 10.1177/09622802251316972

**Published:** 2025-05-14

**Authors:** Kelly Grant, Steven A Julious

**Affiliations:** 1 Norwich Clinical Trials Unit, The University of East Anglia, Norwich, Norfolk, UK; 2 7315 The University of Sheffield, Sheffield, UK

**Keywords:** Cluster randomised, conditional frailty, recurrent events

## Abstract

Recurrent events for many clinical conditions, such as asthma, can indicate poor health outcomes. Recurrent events data are often analysed using statistical methods such as Cox regression or negative binomial regression, suffering event or time information loss. This article re-analyses the preventing and lessening exacerbations of asthma in school-age children associated with a new term (PLEASANT) trial data as a case study, investigating the utility, extending recurrent events survival analysis methods to cluster randomised trials. A conditional frailty model is used, with the frailty term at the general practitioner practice level, accounting for clustering. A rare events bias adjustment is applied if few participants had recurrent events and truncation of small event risk sets is explored, to improve model accuracy. Global and event-specific estimates are presented, alongside a mean cumulative function plot to aid interpretation. The conditional frailty model global results are similar to PLEASANT results, but with greater precision (include time, recurrent events, within-participant dependence, and rare events adjustment). Event-specific results suggest an increasing risk reduction in medical appointments for the intervention group, in September–December 2013, as medical contacts increase over time. The conditional frailty model is recommended when recurrent events are a study outcome for clinical trials, including cluster randomised trials, to help explain changes in event risk over time, assisting clinical interpretation.

## 1 Introduction

Recurrent events are defined as event of the same type, which can occur more than once per person. These re-occurring events can happen when a person has a clinical condition with poor health outcomes they wish to prevent.

Using recurrent events as a study outcome and analysing with commonly used statistical methods such as Cox regression or negative binomial (NB) regression, results in information loss (ignoring time or subsequent events) and inappropriate assumptions such as independence of events. Recurrent events survival analysis methods account for additional information in time (to and between events), recurrent (subsequent) events and within-participant dependence (recurrent events are correlated), for a more accurate approach, utilising the information available more fully, as supported by Ullah et al.,^1^ Yadav et al.,^2^ Thenmozhi et al.,^3^ Yadav et al.^4^ and Watson et al.^5^

Asthma is a condition with recurrent medical appointments. It is a lung condition, often long-term, that can cause breathlessness and wheezing, given by the National Health Service (NHS).^6^ This can be triggered by environmental factors, with treatment aimed at reducing symptoms, such as steroid inhalers. Unmanaged asthma can increase the risk of life-threatening asthma exacerbations, with around 5.4 million people in the UK receiving asthma treatment.^7^ Research by Fleming et al.^8^ and Julious et al.,^9^ indicates peaks in hospital admissions due to asthma difficulties in school-aged children, on return to school after their summer holidays, motivating the preventing and lessening exacerbations of asthma in school-age children associated with a new term (PLEASANT) study^10^ to investigate these peaks.

This article will show how recurrent events survival analysis methods can be extended to cluster randomised trials, accounting for clustering and rare events bias, using PLEASANT trial data as a case study. The analysis is completed alongside simple graphical methods to aid the interpretation of event-specific results, giving new data insights.

## 2 The original analysis

The PLEASANT study investigated the impact of sending a medication reminder letter in July 2013, on reducing unscheduled medical appointments for asthma difficulties, in the September school term. The study was a cluster randomised trial, with general practitioner (GP) practices (clusters) as the unit of randomisation, to the intervention (send reminder letter) or control group.

The primary outcome was the proportion of school-aged (5–16 years) participants with unscheduled medical contact during September 2013, with the aim of reducing the number of events. Secondary outcomes included the number of total medical contacts (unscheduled and scheduled), (steroid inhaler) prescriptions and time to first medical contact, over a range of time periods. Methods of analysis used included a: binary logistic regression model, NB regression model and Cox proportional hazards (shared frailty; random effects to account for clustering by GP practices) model. Model covariates; age (on 1 September 2013), gender, number of medical contacts the previous September (2012), group (intervention and control) and GP practice as a random effect to account for clustering, were also included. Full details of the original methods and the results are given in Supplemental Appendix A.

The study found no evidence of an intervention effect for the proportion of participants with an unscheduled contact in September 2013 (odds ratio (OR): 1.09, 95% confidence interval (CI): 0.96, 1.25), comparable to the number of unscheduled contacts analysis (incidence rate ratio (IRR): 1.02, 95% CI: 0.94, 1.12). However, a 5% reduction in the number of total medical contacts for the intervention group was found in September 2013–August 2014 (IRR: 0.95, 95% CI: 0.91, 0.99), compared to the control group. For the period of September–December 2013, the rate ratio for total contacts is in favour of the intervention group, but statistically non-significant (IRR: 0.96, 95% CI: 0.90, 1.02). Finally, a statistically significant 31% increase in the number of prescriptions collected in August 2013 was found for the intervention group (IRR: 1.31, 95% CI: 1.17, 1.48), compared to the control group.

Key strengths of standard methods used in the original analysis are interpretation ease (incidence rate ratio, percentage change) and model fit (dispersion parameter) of the NB model. However, information loss occurs in discarding time (NB and logistic), recurrent events (logistic and Cox shared frailty) and incomplete GP practice data (NB and logistic). These elements are key for recurrent events analyses to increase statistical power (reduce type 1 error risk), along with; clustering, changing risk of events over time (proportional hazards assumption validity) and within-participant event dependence.

Further limitations include the NB model assuming independent observations, which may be unrealistic for participants with recurrent events. Also, using the Cox survival model assumes event certainty at some point in time (often analyses time to death). However, a medical contact may not be certain to occur. As given by Rodriguez,^11^ this may cause an undefined time to event (improper random variable), where survival density does not integrate to 1. This can be resolved if only participants with medical contacts are included, but invites bias and hazard overestimation. These analysis limitations motivated the work presented in the current paper, using PLEASANT as a case study.

## 3 The analysis aims of the article

The **key research question** of interest for the case study is: when taking into account recurrent events (events of the same type that can occur more than once per participant), does the PLEASANT intervention impact on medical contacts in the September term and are there new insights? The **key aim** is to compare results and conclusions to the original study, investigating if there is additional information when accounting for recurrent events. As the study is a cluster randomised trial, accounting for within GP practice correlation is a priority.

For the PLEASANT study, unscheduled contacts were analysed, along with total (unscheduled and scheduled) contacts and prescriptions. However, Julious et al.^10^ highlighted inconsistencies in how medical contacts were classed as unscheduled (not part of planned care e.g. emergency) or scheduled (planned care), for example, a repeat prescription was classed as unscheduled rather than scheduled if an asthma review was overdue, which inadvertently impacted trial conclusions. Furthermore, far fewer participants have recurrent prescriptions for shorter time periods. So, this case study focuses on **total medical contacts.**

As suggested by Julious et al.,^12^ the primary analysis period of September 2013 may have been too early to detect an intervention effect, with a peak of unscheduled events observed in October/November. Furthermore, there are low proportions of participants with recurrent events for the month-long interval. Hence, it is sensible to focus mainly on **September–December 2013** for this case study, with a higher proportion of participants with recurrent events.

## 4 Methods approach for recurrent events

Recurrent events models can be described using the number of events during intervals (t,t+Δt) , conditional on the event history prior to time *t*, as explained by Cook and Lawless.^13^ Let the number of events in these intervals equal, $\Delta N(t)=N(t+\Delta t^{-})-N(t^{-})$ , and the event history at time *t* equal, H(t)=[N(s):0≤s<t] . Assuming two events cannot occur at the same time, the instantaneous probability of an event at *t*, given the event history, is defined by the following equation:

$$\lambda (t|H(t))=\underset{\Delta t↓0}{\text{lim}}\;Pr\left(\frac{\Delta N(t)=1|H(t)}{\Delta t}\right)$$

This defines the intensity function; the event process for recurrent events, which is analogous to the hazard function for single event processes. The multiplicative intensity model is determined using power laws and can include fixed covariates in a Poisson (counting) process, expressing the intensity as a function of *t* and the covariate history, $x^{\left(t\right)}=[x(u):0\leq u\leq t]$ ,

$$\lambda (t|x^{\left(\infty \right)})=\lambda (t|x^{\left(t\right)})=\lambda_{0}(t)\text{exp}(\beta^{T}Z(t))$$

where $Z(t)=(Z_{1},\ldots ,Z_{p})$ are the *p* model covariates, $\beta^{T}=(\beta_{1},\beta_{2},\ldots ,\beta_{p}{)}^{T}$ are the *p* regression parameters ( p×1 vector), $\lambda_{0}(t)$ is the baseline intensity function for all events. Censored participants have censoring time $c_{ik}$ for the k th event, participant *i*.

Reviewing PLEASANT methodology has highlighted the following key features, that need to be accounted for in a suitable recurrent events analysis approach.


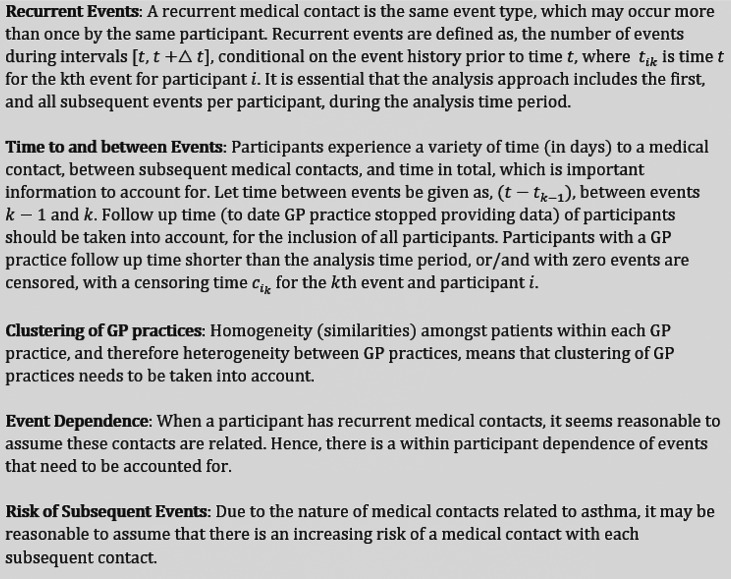



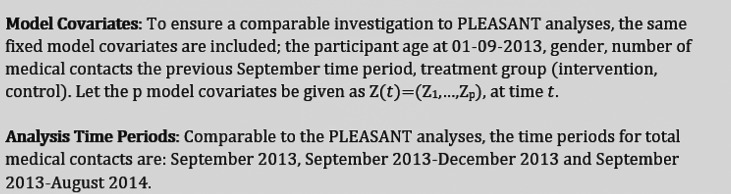


Various Cox proportional hazards model extensions are explored in more detail, to evaluate the best approach for the PLEASANT data, including marginal models, multi-state models, variance corrected models such as Andersen and Gill,^14^ Prentice et al.^15^ total and gap time models, additionally the conditional frailty (CF) model.

## 5 Review of Cox model extensions

To establish and justify the specific statistical model chosen for the recurrent events analysis of the case study, Cox proportional hazard model extensions are explored. Extensions that allow for recurrent events information to be accounted for, as discussed by Kelly and Lim,^16^ differ in their assumptions about:

- the **baseline hazard function**: whether this is the same or event-specific (stratified) for each medical contact (event dependence);
- the **risk set**: whether the participants that are at risk of experiencing a medical contact, just before that particular time point, are unrestricted (same baseline hazard for each medical contact), or restricted (stratified baseline hazard is by event);
- the **risk interval**: whether a participant is at risk of a medical contact during the total analysis time period, or by gap times (time reset after each medical contact);
- the **within-participant correlation/clustering**: whether this is taken into account by using random effects (frailty models) or within the model variance-covariance matrix (variance-corrected models).

The partial likelihood function $\left(L_{p}(\beta )\right)$ for a Cox extension recurrent events model is given by Kelly and Lim^16^ and Liu,^17^ where all events are included to estimate model parameters by maximizing the partial likelihood function. The intensity (hazard) function (λ(t)) , which is the instantaneous event rate, with the key element of being conditional on previous events, as described by Sarkar et al.,^18^ can be replaced by the model-specific intensity function, which adapts the above assumptions for each model,

$$L_{p}(\beta )=\overset{d}{\underset{j=1}{\sum }}\;\frac{\lambda (t_{j})}{\sum_{k\epsilon R(t_{j})}\;\lambda (t_{k})}$$

where λ(t) is the model-specific intensity function, *d* is the observed number of events ordered by time $t_{1}<t_{2}<\cdots <t_{d}$ , $t_{j}$ is the j th ordered event time, $R(t_{i})$ is the risk set of participants at risk at time $t_{i}$ , for the k th event for participant *i*. Censored participants have censoring time $c_{i_{k}}$ for the k th event.

These different model assumptions are attributed to various Cox proportional hazards model extensions, that may result in different conclusions.

### 5.1 Marginal models

The LWA marginal model by Lee et al.^19^ has the same baseline hazard function for all events, using a total time risk interval. However, as given by Kelly and Lim,^16^ a participant is able to be at risk of multiple recurrent events at the same time (rather than conditional on the previous event), which is not acceptable for ordered events (e.g. a participant at risk of a particular event more than once). The WLW marginal model by Wei et al.^20^ has a similar structure to the LWA model, but is stratified by unordered events. So, a participant can be at risk of a later event when an earlier event has not yet occurred, which is also not suitable for recurrent events and may overestimate treatment effects, as highlighted by Kelly and Lim.^16^

### 5.2 Multi-state models

Castaneda and Gerriste^21^ and Amorim and Cai^22^ suggested that multi-state models can analyse a participant transitioning from one state (total contact) to another state (recurrent event) over time. Transition probabilities are calculated between events, to construct intensity functions (which can be estimated using a Prentice, Williams and Peterson (PWP)^15^ model) that represent moving to another event, conditional on a participant experiencing a previous event. There is a lack of research and software development into clustering for multi-state models, and Bijwaard^23^ highlights complexities of how frailty terms could possibly be included.

### 5.3 Variance-corrected models

As explained by Kleinbaum and Klein,^24^ variance-corrected models take into account clustering using robust standard errors, given by White.^25^

#### 5.3.1 Andersen and Gill (AG) model

The AG model by Andersen and Gill^14^ uses counting processes to represent ordered recurrent events over total time. This model has the same baseline hazard function for each event (unrestricted risk set) and assumes events are independent (no event dependence). Clustering can be taken into account using robust standard errors. The AG intensity function offered by Liu^17^ is

$$\lambda_{ik}(t;Z_{ik})=\lambda_{0}(t)\text{exp}(\beta^{T}Z_{ik}(t))$$

where $t_{ik}$ is time t for the kth event for participant *i*, $\lambda_{0}(t)$ is the same baseline hazard for all events at time *t*, $\beta =(\beta_{1},\beta_{2},\ldots ,\beta_{p}{)}^{T}$ are *p* regression coefficients ( p×1 vector), $Z_{ik}=(Z_{1ik},\ldots ,Z_{pik})$ are *p* model covariates (can be time varying) for event *k* for participant *i*.

As discussed by Ullah et al.,^1^ the AG model may be appropriate if the risk of recurrent medical contacts remains constant and there is no within-participant event dependence. However, as offered by Thenmozhi et al.,^3^ within participant recurrent events are likely related. Moreover, research by Suruki et al.^26^ suggests patients with an asthma exacerbation event are at greater risk of a subsequent event, which is increased with frequent previous events or/and more severe asthma. This suggests that within participant event dependence and increased risk of recurrent events need to be accounted for. Additionally, the model covariate of a number of medical contacts in the previous September time period includes information on previous events, but ignores gap time. Hence, the AG model appears unsuitable for the PLEASANT data.

Cook and Lawless^13^ discussed including within participant event dependence as a time-varying covariate within the AG model, such as using ‘previous number of events’, or ‘time since the last event’. However, this would be classed as an ‘internal’ covariate, rather than ‘external’, as it would depend on the recurrent events process (directly influenced by the participant). An external covariate could simply be added to the model (conditioned on the observed covariate values), as the Poisson assumption for a number of events within any interval holds. Whereas for an internal covariate, it would be required to average over the time-varying covariates, modelling these jointly with the recurrent events model. The interpretation of estimations is likely to be difficult and complex.

#### 5.3.2 PWP models

The PWP model by Prentice et al.,^15^ uses an event-specific baseline hazard function (includes event dependence), stratified by ordered event number (restricted risk set). So, a participant is at risk, conditional on their previous event (not at risk until after their previous event). GP practice clustering can be accounted for with robust standard errors. There are two types of PWP models, using total time or gap time risk intervals. The total time model (PWP-TT) uses counting processes to give the hazard of an event for the total analysis time period. Whereas the gap time model (PWP-GT) provides the hazard of an event since the previous event. For both models, all participants are at risk of the first medical contact, but after this, only participants with a previous event are at risk of the next event.

For a total time, the PWP-TT intensity function is offered by Liu^17^ as follows:

$$\lambda_{ik}(t;Z_{ik})=\lambda_{0k}(t)\text{exp}(\beta^{T}Z_{ik}(t))$$

where $\lambda_{0k}(t)$ is an event-specific baseline hazard for the k th event, varying by event number, with all remaining notations as previously defined.

For gap time, the PWP-GT intensity function is described by Liu^17^ as follows:

$$\lambda_{ik}(t;Z_{ik})=\lambda_{0k}(t-t_{k-1})\text{exp}(\beta^{T}Z_{ik}(t))$$

where $\left(t-t_{k-1}\right)$ is the gap time between events k−1 and *k*, with all remaining notation as previously defined.

Taking into account event dependence through stratification by ordered event number, and allowing an increasing risk of within-participant subsequent events, the PWP model appears appropriate for the PLEASANT data. A limitation of the PWP-TT model, as discussed by Kelly and Lim,^16^ is that a potential carryover treatment effect of previous events to subsequent events could be lessened, particularly for much earlier events. Yadav et al.^4^ also highlighted that the PWP-TT model can be more appropriate if the interest is in understanding the covariate effect for the kth event from the study beginning, whereas the PWP-GT model may be more suitable, particularly for asthma data, if the interest is in the *k*th event from the previous event. As the PLEASANT data is specific to asthma data, it may be beneficial to have information on gap times included, for example, in the case of considering a change in medication from one event/exacerbation to the next (rather than purely from the study beginning). So, the gap time model appears most appropriate for these data, taking into account time between events. A further limitation of both PWP models, highlighted by Kelly and Lim,^16^ Amorim and Cai^22^ and Yadav et al.,^2^ is risk sets of later events becoming too small (few participants), causing inaccurate estimates. Kelly and Lim^16^ suggested truncating the data to exclude later event risk sets.

Within the PWP model structure, it is standard to use robust standard errors to include clustering of GP practices. However, previous research around variance-corrected efficiency, by Kelly and Lim^16^ and Box-Steffensmeier and De Boef,^27^ suggests this method may cause under-estimated treatment effects, potentially leading to incorrect statistical inferences, as within-participant correlation is unaccounted for. So, an alternative method for clustering within the model needs to be explored.

### 5.4 CF model

Previous extensive research by Box-Steffensmeier and De Boef,^27^ suggests incorporating a ‘frailty’ term (random effect) into the PWP-GT (gap time) model, to account for participant heterogeneity, instead of using robust standard errors (variance-corrected). This is known as the ‘conditional frailty model’ (CF model). This model is further reviewed by Yadav et al.,^2^ Yadav et al.^4^ and Paudel et al.,^28^ highlighting the advantages in capturing within-participant correlation for recurrent events data. The CF model intensity function is offered by Box-Steffensmeier and De Boef,^27^

$$\lambda_{ik}(t;Z_{ik})=\lambda_{0k}(t-t_{k-1})\text{exp}(\beta^{T}Z_{ik}(t)+w_{i})$$

where $w_{i}$ is a vector of random effects (frailties), for the participant *i*, with all remaining notation as previously defined.

The random frailty term is added within the intensity function, so is at participant level (accounting for individual participant heterogeneity). This intensity function is equivalent to the PWP-GT intensity function, with the frailty term added within this. Estimates for the frailty term are found by maximum likelihood. A global group effect estimate can be given over the analysis time period, or event-specific estimates. Paudel et al.^28^ adapted the CF model to use a group-specific fixed effect for group-level heterogeneity, in addition to including the frailty term for individual heterogeneity. In the case of accounting for clustering by GP practice, it seems highly appropriate to use the **frailty term at the GP practice level** (as do other standard models such as the Cox shared frailty model (used in the PLEASANT study), given by Balan and Putter^29^), rather than individual level. So, each GP practice shares a random effect amongst its patients, replacing $+w_{i}$ with $+w_{g}$ , within the CF model intensity function, for the participant *i* in the cluster *g*.

Therefore, using the frailty term to account for GP practice clustering, event-specific baseline hazards to account for event dependence and increasing risk of subsequent events, as well as taking into account time between events, this model appears to meet all PLEASANT data requirements.

## 6 Recurrent events methodology summary

Table 1 summarises if the reviewed statistical approaches include elements of the model requirements; recurrent events, time, GP practice clustering, within-participant event dependence and increasing risk of subsequent events.

**Table 1.** PLEASANT data recurrent events requirements included by model.

	Recurrent			Event	Increasing
Model	events	Time	Clustering	dependence	event risk
Binary logistic	No	No	Yes	No	No
NB	Yes	No	Yes	No	No
Cox shared frailty	No	Yes	Yes	No	No
AG	Yes	Yes	Yes	No	No
PWP-TT	Yes	Yes	Yes	Yes	Yes
PWP-GT	Yes	Yes	Yes	Yes	Yes
Marginal	Yes	Yes	Yes	No	No
Multi-state	Yes	Yes	No	Yes	Yes
Conditional frailty	Yes	Yes	Yes	Yes	Yes
Standard frailty	Yes	Yes	Yes	No	No

PLEASANT: preventing and lessening exacerbations of asthma in school-age children associated with a new term; AG: Andersen and Gill; PWP-TT: Prentice, Williams and Peterson with total time model; PWP-GT: Prentice, Williams and Peterson with gap time model.

Inadequacies of the reviewed statistical models to investigate recurrent events are lack of recurrent events (logistic and Cox shared frailty), lack of time (logistic and NB), or time to first event only (Cox shared frailty), or lack of gap times (all models except PWP-GT and multi-state), lack of event dependence and increasing risk of subsequent events (logistic, NB, Cox shared frailty, AG, marginal and standard frailty). Moreover, there are issues of unreliable estimates using robust standard errors (variance-corrected models; AG, PWP and marginal), lack of software development for clustering (multi-state), unordered events (marginal) and a lessening event-to-event carryover effect (PWP-TT). These model limitations could create model misspecification, causing under or over (inaccurate) estimations of the treatment effect, leading to incorrect statistical inferences.

However, the CF model (PWP-GT model plus frailty term) accounts for all model requirements and are the most appropriate model to investigate recurrent events within the PLEASANT data. Testing for event dependence and clustering, whilst considering rare events bias and completing sensitivity analyses of truncating later event risk sets, is a sensible approach.

### 6.1 Software available

R software available for the variance-corrected and marginal models includes the survival package, using the ‘coxph’ function, by Therneau.^30^ Time-dependent covariates can be used in conjunction with the ‘rms’ package; ‘cph’ function, by Harrell.^31^ Multi-state models can be investigated using the ‘msm’ package, by Jackson^32^ in R. SAS provides the PHREG procedure for Cox model extensions and NLMIXED for the frailty random effects term. Paes and Lima^33^ developed the PTRANSIT macro that can be used for multi-state modelling in SAS. Stata provides survival analysis commands including STSET for data preparation, and STCOX to fit the relevant Cox extension model. Crowther and Lambert^34^ developed the MULTISTATE Stata package, which includes the MSSET command for multi-state data preparation, as well as STMS and PREDICTMS for multi-state modelling and predictions.

## 7 CF model considerations

### 7.1 Choice of frailty distribution

There is a lack of research in distribution choice for the frailty random effects term. However, a gamma distribution is the standard choice, for ease of application in modelling a positive random variable, as suggested by Kelly and Lim,^16^ Bijwaard,^23^ Yadav et al.,^2^ Yadav et al.^4^ and Paudel et al.^28^ This is due to the unconditional survival and hazard function closed form expressions being straight-forward to derive, the flexibility of the distribution and relatively low computational demand, compared to other distributions. Balan et al.^29^ and Bijwaard^23^ discussed alternative options of frailty distribution choices, such as the log-normal distribution. It is suggested that the likelihood ratio test (LRT) could be used to select the most suitable frailty distribution, however, this may not be ideal as the frailty terms are latent, so it can be difficult to assess goodness-of-fit from the data. Hence, using a gamma frailty distribution is most sensible, whilst providing a comparison using a log-normal distribution as a sensitivity analysis.

### 7.2 Rare events bias adjustment

The primary population of 11,564 participants is large, but if few participants have multiple events, recurrent events can be considered rare, potentially causing inaccurate estimates. So, adjustments for a ‘rare events bias’ may be required, as recommended by Box-Steffensmeier and De Boef.^27^ Lin et al.^35^ investigated and recommended using Firth's penalised likelihood for Cox models when the number of events is small, to reduce bias and variability in parameter estimates. As offered by Lin et al.,^35^ Firth's approach is given by the following equation:

$$L_{p}*(\beta )=L_{p}(\beta )|I(\beta ){|}^{\frac{1}{2}}$$

where $\hat{\beta }=\text{argmax}\left\{L_{p}(\beta ){\left|I(\beta )\right|}^{\frac{1}{2}}\right\}$ is the Firth estimate (maximum of penalised likelihood $L_{p}*(\beta )$ ), $L_{p}(\beta )$ is the CF model partial likelihood function, I(β) is the Fisher information matrix (contains coefficient variability).

Lin et al.^35^ discussed a minimum ‘rule of thumb’ of 10 events per predictor variable (EPV), to minimise coefficient estimate bias and variability. Their findings suggest greater accuracy using Firth's penalised likelihood, particularly for models with categorical predictors, when the EPV is equal to or fewer than six. The CF model for PLEASANT contains four fixed effects, which include two categorical predictors (group and gender) and random effects for the frailty term. This equates to 5×10=50 ‘events’ as a minimum, or using Lin's et al.^35^ findings, 5×6=30 ‘events’. For a recurrent events analysis, this may translate to a minimum of 30–50 participants with multiple events. However, with the complexity of stratified event risk sets, a more cautious approach may be preferred, using Firth's penalised likelihood (comparison to the partial likelihood) if fewer than around 100 participants have multiple events.

### 7.3 Truncation of data

As discussed, later event risk sets may be too small for reliable model estimates, with a solution of data truncation to exclude these. Reviewing previous research, there is no clear guidance on truncation cut-off points for the CF model. However, taking into account the rare events bias adjustment methodology, a small events risk set could be considered to be fewer than 30–50 participants for PLEASANT data, according to Lin et al.^35^ findings. Due to the complex strata structure, it is worth investigating a range of truncation points, excluding later event strata of fewer than around 30–500 participants. Subsequently, comparing consistency in effect direction and size, associated CI width and statistical significance, as sensitivity analyses. Based on this methodology, a choice of **five truncation points** include event risk sets above equivalent proportions and counts of the primary population:

1. >5% of the primary population, > 578 participants,
2. >2% of the primary population, > 231 participants,
3. >1% of the primary population, > 115 participants,
4. >0.5% of the primary population, > 57 participants,
5. >0.25% of the primary population, > 28 participants.

### 7.4 Diagnostic testing

If there is uncertainty about whether event dependence or clustering exists, it may be difficult to determine if event stratification or/and a frailty term is needed. Box-Steffensmeier and De Boef^27^ suggested using the following diagnostics.

To test for evidence of event dependence, a cumulative hazard function plot (by event number), should indicate if baseline hazards vary from event to event. If event dependence is not justified, the nested AG plus frailty term model may be preferred, known as the ‘standard frailty model’ by Box-Steffensmeier and De Boef^27^ and Yadav et al.^4^ Incorporating event dependence (stratify by event number) when this cannot be justified, would potentially increase model complexity unnecessarily, leading to overfitting. However, ignoring event dependence when this is justified, assumes common baseline hazards, losing this information and increasing bias (Hernández-Herrera et al.).^36^

As supported by Balan and Putter^29^, to test for evidence of individual heterogeneity (clustering in this case), the LRT may be used to test the statistical significance of the frailty term. If the frailty term is statistically non-significant, this may suggest using the nested PWP-GT model (no frailty term). Using statistical tests to determine the appropriate method to analyse data, means reliance is based on the p -value result to choose the ‘appropriate’ method. This should be done with caution for non-statistically significant results. This is because in this case, the LRT would be used to determine whether or not to include the random effects frailty term, which is reasonable as a check to account for clustering. Thus, if the frailty term is deemed necessary by the LRT, but shows a negligible level of clustering in the final model, there may be little difference in model estimates, with or without the frailty term. However, if the frailty term is deemed unnecessary by the LRT, but clustering is actually present, this would be unaccounted for.

The LRT for nested models assumes that the LRT test statistic asymptotically converges to the chi-squared distribution, as the sample size increases. This seems plausible for the PLEASANT data, as the sample size is large. For these recurrent events data, it is worth noting a deviation from LRT assumptions of independent observations (recurrent events are assumed dependent within the participant), which is not necessarily required for the Wald test, which could be used as a comparison test.

Using various statistical tests can present issues of multiplicity. Multiple testing can inflate the type I error, potentially leading to false-positive conclusions, so approaches to appropriately adjust the error level should be considered. However, the LRT (or Wald test) here is used for model selection only, rather than answering the research question, so adjusting the error level is unnecessary in this case.

### 7.5 Mean cumulative function

To aid interpretation of event-specific results, a mean cumulative function (MCF) plot is used. The event rate by group can be plotted over time, to visualize any group difference. This plots the sample non-parametric MCF, also known as the Nelson-Aalen estimator of the cumulative hazard rate function. It is given by Hobbs^37^ as follows:

$$\hat{\Lambda }(t)=\int_{0}^{t}\;\frac{J(u)}{Y.(u)}\text{d}N\cdot (u),\;0\leq t\leq \tau$$

Where recurrent events data for *N* participants are within a finite interval of [0,τ] , $T_{i}$ are the event times, $U_{i}$ are the censored times, then $N_{i}=[N_{i}(t),t\geq 0]$ is the observed counting process for participant *i*. For $[Y_{i}(t),t\geq 0],Y_{i}(t)=1$ if and only if the i th participant is uncensored and survived at time $t^{-}$ .

1.
$F^{-}=\sigma [N_{i}(u),Y_{i}(u),i=1,\ldots ,n;\;0\leq u\leq t]$ is the filtration up to *t* (not including t ), for each t>0
2.
$N\cdot (t)=\sum_{i=1}^{n}N_{i}(t)$ are the counting processes for the total number of events in (0,t]
3.
$Y\cdot (t)=\sum_{i=1}^{n}Y_{i}(t)$ are the total number at risk in (0,t]
4.
$J(t)=I_{\left(Y\cdot (t)>0\right)}$ indicates whether at least one participant is at risk at the time t

## 8 Recurrent events analysis results

CF model data must be prepared, and ordered by participant and event (date order). ‘Start’ and ‘stop’ (gap) times are calculated in days from 01/09/2013 (study start) to the first event (all participants start at zero), then from the first to the second event (and so on) for each participant within the analysis time period, used as ‘survival times.’ Events are counted cumulatively per participant, and used as strata. Follow-up time (to date GP practice stopped providing data) of participants is taken into account, for the inclusion of all participants. Participants with a GP practice follow-up time shorter than the analysis time period, or/and with zero events are censored. This data structure follows guidance from Castaneda and Gerriste,^21^ Thenmozhi et al.^3^ and Therneau,^30^ with an example (fictitious data) given in Supplemental Appendix B1. The final model example code used in R^38^ is given in Supplemental Appendix B2. Analyses are performed using R package ‘survival’^39,40^ and ‘coxphf’.^41^

Table 2 gives counts and proportions of participants with the maximum number of total (unscheduled and scheduled) medical contacts. Each time period shows a reasonable number of participants with recurrent events (September 2013: 2924 (25.3%), September–December 2013: 7908 (68.4%) and September 2013–August 2014: 9686 (83.8%)), suggesting a rare events adjustment is unnecessary.

**Table 2.** Number and proportion of participants with a maximum of zero, one and multiple total contacts, for each time period, out of 11,564 participants.

	Maximum per participant			Total
Time period	0 events	1 event	Multiple events	Events
Sep 13	5792 (50.1%)	2848 (24.6%)	2924 (25.3%)	11,711
Sep–Dec 13	2304 (19.9%)	1352 (11.7%)	7908 (68.4%)	47,146
Sep 13–Aug 14	1362 (11.8%)	516 (4.5%)	9686 (83.8%)	120,052

To assess event dependence, cumulative hazard plots for each time period (Appendix C, Figures 3.1 to 3.3 of Supplemental Material) show baseline hazards vary by event number, with increasing risk of subsequent events, justifying model event stratification for total contacts data.

To test for clustering, Table 3 shows statistically significant results in the LRT for the frailty term, for each time period, justifying the presence of clustering for total medical contacts data. Albeit the frailty term variance appears fairly low (September 2013: 0.0314, September–December 2013: 0.0114, September 2013–August 2014: 0.0061), perhaps indicating a low level of between GP practice variability.

**Table 3.** Frailty term (total contacts conditional frailty model) results for each time period, using the likelihood ratio test (LRT), Wald test and variance.

Time period	LRT *p*-value	Wald test *p*-value	Variance
Sep 13	<0.001	<0.001	0.0314
Sep–Dec 13	<0.001	<0.001	0.0114
Sep 13–Aug 14	<0.001	<0.001	0.0061

CF model results for total medical contacts, are given in Table 4, with global estimates of the effect. There is no evidence (*p *> 0.05) of a difference in risk of a total contact between groups at a particular time point for September 2013 (intensity ratio (IR): 0.989, 95% CI: 0.918, 1.064, *p *= 0.758), September–December 2013 (IR: 0.971, 95% CI: 0.931, 1.013, *p *= 0.172), or September 2013–August 2014 (IR: 0.977, 95% CI: 0.948, 1.008, *p *= 0.141). All intensity ratios are in favour of the intervention group, showing a statistically non-significant 1%–3% risk reduction in total contacts, compared to the control group.

**Table 4.** Conditional frailty model results for group allocation (total contacts), in favour of the intervention group (IR < 1).

Time period	IR	95% CI	*p*-value
Sep 13	0.989	(0.918, 1.064)	0.758
Sep–Dec 13	0.971	(0.931, 1.013)	0.172
Sep 13–Aug 14	0.977	(0.948, 1.008)	0.141

Model covariates include participant age on 01-09-2013, gender, number of medical contacts in the previous time period, and treatment group.

Sensitivity analysis results investigating truncation points for total contacts to improve accuracy, as well as results using a log-normal distribution for the frailty term, are given in Supplemental Appendix D. These show overall consistency in effect size, direction, CI width and statistical significance. In terms of frailty term variance, the log-normal distribution results suggest a similar, albeit marginally lower level of between GP practice heterogeneity.

Focusing on September–December 2013 for total contacts, event-specific results are explored. Later event risk sets are small, so to promote accuracy, these are truncated to 12 events maximum, containing 5% of the total population or more.

**Figure 1.**
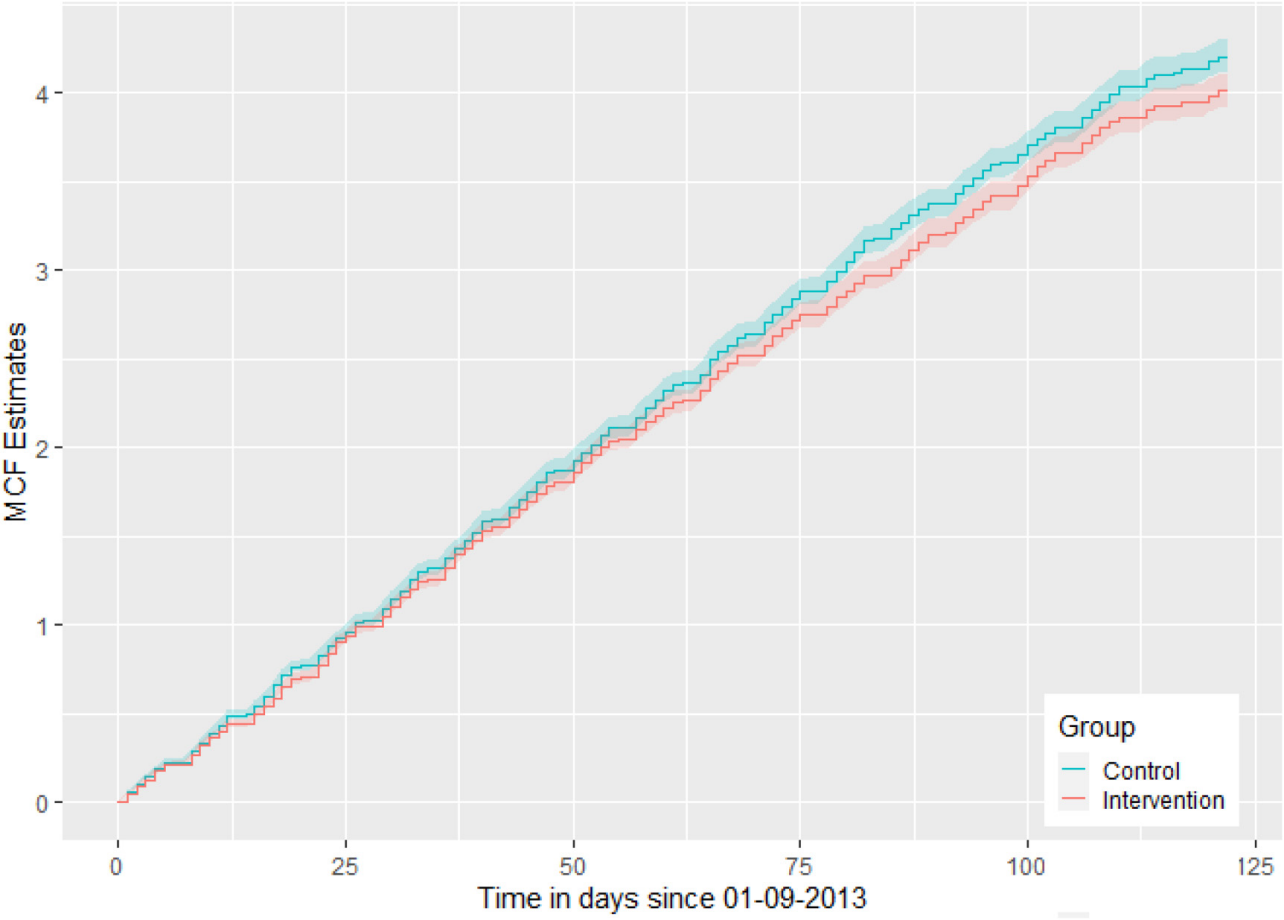
Mean cumulative function plot by group, for total medical contacts, over September–December 2013.

Figure 1 gives the MCF plot; a non-parametric analysis of recurrent events by group, using the Nelson-Aalen estimator of the cumulative hazard rate function, as described and supported by Hobbs.^37^ It shows that the cumulative number of total medical contacts over time is less for the intervention group, compared to the control group, for later events. The event rate over time is fairly constant for both groups and lessens for later events, but more so for the intervention group from around 60 days (beginning November 2013), with increasing visible group difference until the end of December 2013. The MCF is obtained using the R package ‘reda’.^42^

Table 5 shows the number of participants censored (participants censored at zero days are those with zero events, whereas participants censored during the remaining 122 days are due to GP practices stopping providing data), the cumulative number of events and the number of participants at risk for each event number, by group (I = intervention, C = control). As a participant is not at risk of an event until after their previous event, the number at risk is conditional on the previous event. The majority of participants that are experiencing events have these occur earlier in the analysis period, mostly around 30 days (end September/beginning October), compared to later in the interval, with few participants censored for missing GP practice data.

**Table 5.** Number at risk by event number, by group (I = intervention, C = control), including number censored and cumulative events, for total events Sep–Dec 2013.

	At risk	Censored		Cumulative number of events at				Censored
Event/group	0 days	0 days	30 days	30 days	60 days	90 days	122 days	1–122 days
1/I	5631	1110	0	2897	3893	4331	4521	36
1/C	5933	1194	4	3026	4079	4529	4739	50
2/I	4485	0	474	2926	3599	3801	3833	46
2/C	4689	0	564	3145	3846	4039	4075	31
3/I	3787	0	378	2500	3010	3108	3118	23
3/C	4044	0	480	2753	3262	3372	3384	26
4/I	3095	0	316	2056	2407	2457	2459	15
4/C	3358	0	419	2310	2653	2706	2708	9
5/I	2444	0	272	1679	1890	1908	1909	7
5/C	2699	0	362	1909	2145	2157	2157	7
6/I	1902	0	219	1336	1452	1459	1459	6
6/C	2150	0	282	1518	1657	1673	1673	5
7/I	1453	0	198	1041	1113	1116	1116	1
7/C	1668	0	255	1198	1280	1291	1291	5
8/I	1115	0	174	832	878	879	879	0
8/C	1286	0	192	919	964	967	967	1
9/I	879	0	127	657	687	688	688	2
9/C	966	0	160	698	730	733	733	0
10/I	686	0	110	489	508	510	510	0
10/C	733	0	148	554	562	563	563	1
11/I	510	0	77	373	387	387	387	2
11/C	562	0	104	410	423	423	423	0
12/I	385	0	55	293	301	301	301	0
12/C	423	0	84	325	333	333	333	0

**Event/group**: Event number (1–12)/intervention (I) or control group (C). **At risk**: A participant is not at risk of an event until after their previous event, so the number at risk is conditional on the previous event. **Censored**: Participants censored at 0 days are those with zero events, whereas those censored during the remaining 122 days are due to GP practices stopping providing data. **Cumulative number of events**: the sum of all participant events experienced up to X days, for each event number and group.

The CF model results given in Table 4 give global estimates of the intensity ratio for the group effect; an overall effect estimate (statistically non-significant 2.9% risk reduction in total contacts, compared to the control group, for September–December 2013). Table 6 presents the event-specific results (event risk sets truncated to and presented to 12 events, where event risk sets contain 5% of the total population or above), where the IRs depend on each of the strata (each event number has a different baseline intensity) and the treatment group. Event-specific IRs are mostly in favour of the intervention group (except for events 1 and 6 with an IR slightly over 1, but are statistically non-significant, with CIs including 1), consistent with the global estimate (IR: 0.971, 95% CI: 0.931, 1.013). The IRs for events 4, 10, 11 and 12 are statistically significant (*p *= 0.034, *p *= 0.030, *p *= 0.040 and *p *= 0.006, respectively) and decrease from 0.931 at event 4, to 0.798 at event 12, suggesting a 6.9%–20.2% risk reduction in total contacts for the intervention group for later events, compared to the control group, during September–December 2013. There is also weak evidence (*p *= 0.052) of a 6.9% risk reduction for event 5.

**Table 6.** Conditional frailty model event-specific results for group allocation (total contacts), truncated to 12 events, mostly in favour of the intervention group (IR < 1).

Event number	Intensity ratio (IR)	95% CI	*p*-value
1	1.040	0.983, 1.099	0.170
2	0.987	0.932, 1.046	0.670
3	0.954	0.897, 1.015	0.134
4	0.931	0.871, 0.995	0.034
5	0.931	0.865, 1.001	0.052
6	1.005	0.928, 1.089	0.898
7	0.985	0.901, 1.076	0.737
8	0.993	0.899, 1.097	0.896
9	0.969	0.867, 1.083	0.574
10	0.869	0.765, 0.986	0.030
11	0.860	0.745, 0.993	0.040
12	0.798	0.679, 0.938	0.006

Model covariates include participant age on 01-09-2013, gender, number of medical contacts during the previous time period, and treatment group.

An example of the R code^38^ for this model, giving event-specific results, is given in Supplemental Appendix B3, using the R package ‘survival’.^39,40^ This model formula code approach for event-specific results is used and supported by Abreu and Sousa-Ferreira.^43^

**Figure 2.**
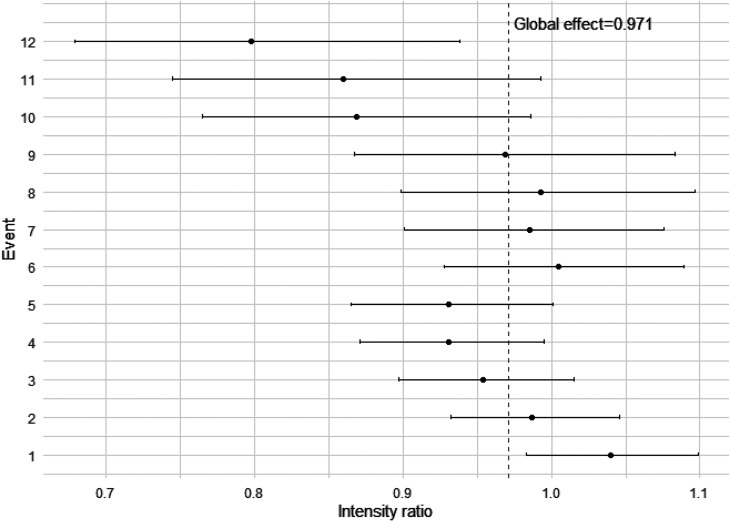
Conditional frailty event-specific intensity ratios for group allocation, truncated to 12 events, for total contacts during September–December 2013 (dashed line is the global effect of 0.971).

Figure 2 shows a forest plot of the event-specific results for total medical contacts during September–December 2013, to combine these results visually. The event-specific IRs and CIs appear to cluster around the global effect of 0.971 (dashed line) for the first nine events, then events 10–12 show more of a group difference, in favour of the intervention group, corresponding to the MCF conclusions. CIs appear to widen as the event number increases. A rationale for this could be due to small strata samples experiencing a large number of events.

Supplemental Appendix E gives the CF model analysis results for unscheduled medical contacts, where no evidence of an intervention effect is found for each analysis period, matching the original study conclusions. Consistency is shown in the sensitivity analysis results (Supplemental Appendix F) and conclusions, in terms of effect size, direction, uncertainty and statistical significance.

Supplemental Appendix G gives CF model results and conclusions for the prescription data. The recurrent events analysis finds strong evidence to suggest the intervention group experienced a 29.6% risk increase in prescriptions (uptake), at any particular time point during August 2013, compared to the control group. This statistically significant result closely matches the original study's conclusion and aim of increasing prescription collection during the summer. Sensitivity analyses investigating truncated data for prescriptions (Supplemental Appendix H), show consistency in results for effect size, direction, CI width and statistical significance for the time period August 2013–July 2014, as well as when using a rare events bias adjustment for August 2013 and August 2014. This is due to few participants experiencing recurrent events in August 2013 and August 2014, so using a rare events bias adjustment and truncating to two prescriptions events is appropriate for these data, to improve accuracy.

## 9 Discussion

The CF model seems an effective way of analysing recurrent events data, that can be extended to cluster randomised trials by using the frailty term at the cluster level, with the options of truncation of small event risk sets and adjusting for rare events bias, to improve the accuracy of estimates. Visualising results using the MCF and a forest plot aids event-specific results interpretation and provides important data insights, further to the global effect. Using an MCF plot to visualise recurrent events for chronic conditions is recommended by Phillips et al.^44^

Comparing the recurrent events analysis of total contacts to the standard PLEASANT analyses, overall the results appear most similar to the NB results (September 2013 IRR: 0.966, September–December 2013 IRR: 0.955, September 2013–August 2014 IRR: 0.949). The NB rate ratios are slightly smaller than the recurrent (global estimation) IRs (September 2013: IR: 0.989, September–December 2013 IR: 0.971 and September 2013–August 2014 IR: 0.977), with CIs up to 1.5 times larger. However, both analyses show effects are in favour of the intervention group. Specifically for September–December 2013, a statistically non-significant reduction in total contacts of 4.5% (NB) and risk reduction of 2.9% (CF) is found, compared to the control group. In terms of statistical significance, the NB result for September 2013–August 2014 is significant (*p *= 0.025), but non-significant for the recurrent events analysis. Overall, the recurrent events analysis shows higher precision (narrower CIs).

Focusing on the September–December 2013 period for total contacts, the event-specific results for the CF model are consistent with the CF global estimate, in terms of most IRs (except events 1 and 6, with IRs slightly above 1, although statistically non-significant, with CIs including 1) in favour of the intervention group, with slightly wider CIs. The statistically significant IRs for events 4, 10, 11 and 12 reduce from 0.931 to 0.798, which could suggest that the more medical contacts a participant has, the larger the risk reduction is over time for those in the intervention group, compared to the control group. This conclusion is supported by the MCF plot (Figure 1). This result perhaps highlights the importance of sending the summer medication reminder letter to participants with multiple (such as four or more) total medical contacts during September–December.

It is worth noting that the event-specific analyses do not include any multiple comparison corrections, such as the Bonferroni correction (based on a repeated sampling method by Neyman and Pearson^45^). There are a limited number of event-specific results, so it seems appropriate to avoid multiple comparison corrections in this case. This does potentially increase the risk of a false-positive result, however, employing multiple comparison corrections, which may be conservative for these limited number of results (e.g. a Bonferroni-adjusted significance level of 0.05/12 = 0.004, or 0.4%, for data truncated to 12 events) would reduce the statistical power and may make it more difficult to detect any true differences between groups. Perneger^46^ suggests that multiple comparison correction may be appropriate when investigating for associations, using multiple tests such as unplanned subgroup analyses (repeated sampling), without pre-determined hypotheses. Whereas these analyses are planned and limited to event-specific, so simply explaining our methods, results and discussion points should ensure the reader has adequate information for interpretation, without the need for multiple comparison corrections.

However, when investigating event-specific results, it may be important to consider bias. As Aalen et al.^47^ describe, recurrent events bias could arise from sub-populations with different event risks; treatment-resistant (high event risk) and treatment-effective (low event risk) populations, both within the intervention and control groups. If the treatment-effective population reduces in the intervention group due to receiving effective treatment, leaving mostly or only the treatment-resistant population, compared to a mixture in the control group, this may create unreliable event-specific estimates and caution is needed for interpretation. Aalen et al.^47^ explained that if this individual heterogeneity is unaccounted for, this bias could cause Simpson's paradox over time, where the group intensity functions can ‘artificially’ cross over, so the treatment may appear to have a (misleading) harmful effect compared to the control for later events.

As an alternative to bias or randomness, event-specific results showing a larger effect for later events could also be influenced by clinical reasons. For example, the more unwell or severe the patients’ asthma, the more events they may have, compared to patients with less severe asthma, creating a sub-population that may have different outcomes in later events. This sub-population could be treated effectively in the intervention group, leaving a mixture in the control group, impacting the larger effect seen for later events.

Despite raised concerns of bias for recurrent events analyses, the CF model incorporates a frailty term to account for participant heterogeneity, which may help reduce these biases. Truncating the event risk sets to only those that contain at least 5% of the total population, is performed to also reduce bias. Furthermore, the option of presenting an overall global estimate or/and event-specific estimates is available. Event-specific estimates of the group effect can be advantageable, to observe how risk may change over time/number of events (perhaps a better representation of clinical reality). The model does assume that the other covariates have the same estimates for each event number, where a further alternative approach could be to use time-varying covariates, for a more tailored modelling approach over time.

Truncating data can raise concerns, over information loss. However, 100% of participants are included, only the number of events per participant is capped, aimed at preventing small risk sets from skewing results. If a small sample of participants have recurrent events, a rare event adjustment can also reduce bias. These are important features for the prescriptions data (Supplemental Appendix G), as few participants collect multiple prescriptions during the month-long periods, resulting in narrower CIs for the effect estimate.

Considering the model choice of PLEASANT and this article, the NB analysis (most similar global results) includes subsequent events, however, time is discarded and observations are assumed independent (unreasonable for recurrent events), therefore events per participant are assumed evenly spaced. Whereas time between recurrent events (gap time) is included in the recurrent analysis, so participant patterns of events are accounted for. Also, GP practices with incomplete data for each time period are excluded from the NB analysis, but included in the recurrent events analysis. An alternative NB method could be to use an offset of the logarithm of GP practice follow-up time for each participant, to include all GP practices, but lack of event gap time is still an issue. Furthermore, the CF model includes event stratification and a frailty term (rather than robust standard errors which can underestimate treatment effects), so within participant event dependence, increasing risk of subsequent events and clustering is accounted for (the NB model assumes constant risk). The frailty term variance appears low in each case (perhaps indicating a low level of between GP practice variability), however, is above zero and statistically significant, suggesting clustering is present in these data. Using a log-normal distribution for the frailty term, instead of a gamma distribution, still justifies the use of the frailty term for clustering within these data and overall, produces consistent CF results and conclusions. These model additions increase model complexity, but are validated simply using diagnostics.

It is crucial to consider the interpretation of effect estimates, where IRs (CF) compare the risk of an event by group, at any particular time point, whereas, rate ratios (NB) are essentially a ratio of two group means, given as a percentage change. Arguably, rate ratios are easier to interpret, with a simple explanation of group differences. However, IRs account for time, which is intuitive and all-encompassing in interpretation.

Despite the similar results in estimates of the NB and CF analyses, a statistical power increase for the CF model is plausible, due to the extra information included and consequent increase in precision. For recurrent events studies where the true treatment effect is small (or/and clustering variation is high), this extra statistical precision (and appropriate CF model assumptions) could be essential in detecting this effect as statistically significant and determining correct statistical inferences (avoiding model misspecification).

## 10 Further work and development

There appears to be no current guidance on recommended truncation points, or when to use a rare events adjustment, for the CF model. Further research could help determine statistical rules for the minimum recommended count and proportion of participants within an event risk set (for truncation) and minimum with multiple events (rare events adjustment), to improve model accuracy. Research by Paudel et al.^28^ shows development in adapting the model to account for both individual and group-level heterogeneity. However, there is a lack of research in using the CF model frailty term for clustering, which works well for PLEASANT data (satisfies model assumptions).

The number of CF model event risk sets can be large, so presenting ‘number at risk’ results for recurrent events can present difficulties, which could be a development area. Study design such as sample size formula (currently no proposed analytic formula) needs considering for the CF model. With extra information on recurrent events, time, clustering, event dependence and increasing risk of subsequent events, compared to standard methods, a gain in precision and consequent statistical power increase is likely, particularly as the number of events increases (more information). This precision increase is supported by narrower intensity ratio CIs, as the CI width of the estimate is closely related to sample size, given by Cook et al.^48^ and Liu.^49^

Research by Jahn-Eimermacher et al.^50^ and Tang and Fitzpatrick^51^ explores the sample size calculations for recurrent events using the AG model and frailty terms. Jahn-Eimermacher et al.^50^ suggest sample size (and power) is sensitive to censoring and within-participant correlation (frailty term). However, the AG model has different statistical assumptions of constant event rates and total time (no event dependence), compared to the CF model. Censoring is higher when fewer participants have events and (for PLEASANT) with incomplete GP practice data, potentially increasing sample size. Frailty term variance is small for PLEASANT, suggesting less effect on sample size.

Considering sample sizes for cluster randomised trials, it may be important to consider a definition of intracluster correlation for recurrent events, however, there appears to be a lack of research in this area, which may require further development.

In future studies using routine data, a more specific definition of unscheduled contacts could be explored, such as unscheduled hospitalisations or GP emergency appointments for asthma exacerbations only. Alternative interventions could include medication reminder text messaging to participants, further reducing the cost of time and resources for GP practices and the NHS.

## 11 Conclusions

Using the CF model with the frailty term at the cluster level, plus a rare events bias adjustment and truncation of small event risk sets as necessary, alongside the MCF, provides a practical and efficient approach to analysing recurrent events in a cluster randomised trial. Model assumptions are assessed through simple diagnostics and results are interpreted effectively using graphical methods, visualising additional data insights at an event-specific and global effect level.

Comparing the CF model recurrent events analyses to the standard PLEASANT analyses, overall results are most similar to the NB model results. It could be argued that incidence rate ratios are simpler to interpret, compared to the risk increase/reduction interpretation using the CF model intensity ratios. However, the recurrent events analysis has the clear advantage of capturing extra information on time, within-participant event dependence, increasing risk of subsequent events, and a more inclusive approach for clustering and information from all GP practices. This creates a more holistic analysis, limiting information loss, potentially increasing statistical power (supported by narrower IR CIs) and improving accuracy by truncating small event risk sets and using a rare events adjustment. Furthermore, providing global and/or event-specific estimates of the effect gives the option of assessing how risk may change over time/number of events. This may provide a better representation of clinical reality, compared to the NB model which assumes a constant risk over time. Observing risk over time may highlight a particular number of events where risk increases and it is deemed statistically and/or clinically necessary for particular clinical input, for example.

The final conclusions for the recurrent events analysis for total medical contacts (global estimates) are consistent with the NB model results, in the direction (in favour of the intervention group) of the effect, but with narrower CIs (higher precision). For September–December 2013, the original study found a statistically non-significant reduction in total contacts of 4.5% (NB), whilst the global estimate of the CF model suggests a statistically non-significant risk reduction of 2.9% in total contacts, compared to the control group.

The event-specific results are consistent with the global estimate, with most intensity ratios in favour of the intervention group and statistically significant for events 4, 10, 11 and 12. The results suggest a risk reduction in total medical contacts, increasing from around 6.9% to 20.2% for the intervention group, as the number of events increases, compared to the control group. This may highlight a larger (and increasing) intervention effect for participants with more (at least 4) total medical contacts during September–December. Concerns around bias for event-specific estimates may be reduced by using the frailty term within the CF model, along with truncation of small event risk sets.

Based on the recurrent events analysis sensitivity results, guidelines could suggest to use of a rare events bias adjustment for the CF model, if less than around 1% of the total participant population have recurrent events, or if less than around 20 participants have recurrent events per predictor variable. This is based on a cautious approach due to the stratified model structure, using PLEASANT data results, whilst considering the ‘rule of thumb’ of 10 events per predictor variable, as discussed by Lin et al.^35^ Further to this, particularly when using event-specific results, it seems highly sensible to use truncation of event risk sets for the CF model if there are risk sets that include less than around 5% of the population, or risk sets of less than around 100 participants per predictor variable, to promote accuracy.

Further research into truncation points, rare events adjustment, clustering, time-varying covariates, presenting results and sample size calculation could benefit the development of recurrent events analysis, using the CF model.

## 12 Recommendations

Recurrent event survival analysis methods are recommended when there are recurrent events for a study outcome, for clinical conditions such as asthma, potentially increasing statistical power by including extra information (time, recurrent events and within-participant dependence), leading to higher precision compared to standard methods. Furthermore, the CF model can give a global estimate of the effect, as well as event-specific results, depending on whether the interest is overall, or/and to assess risk over time/number of events, which is a clear methodology advantage for clinical interpretation. It is recommended to use the MCF plot, alongside the CF model event-specific and global results, to aid interpretation and visualise results over time, plus truncation of small event risk sets and a rare event bias adjustment, where necessary, to improve model accuracy.

Use of the frailty term at cluster level within the CF model is recommended to account for clustering, and to extend recurrent events analysis methods to cluster randomised trials.

## Supplemental Material

sj-docx-1-smm-10.1177_09622802251316972 - Supplemental material for Analysis of recurrent events in cluster randomised trials: The PLEASANT trial case study Supplemental material, sj-docx-1-smm-10.1177_09622802251316972 for Analysis of recurrent events in cluster randomised trials: The PLEASANT trial case study by Kelly Grant and Steven A Julious in Medical Research
