# Supplementary material for: Analysis of recurrent events in cluster randomised trials: The PLEASANT trial case study
Source: Stat Methods Med Res. 2025 May 14;34(6):1079–96. doi: 10.1177/09622802251316972 (PMC12209553; doi:10.1177/09622802251316972)
Supplement: sj-docx-1-smm-10.1177_09622802251316972 - Supplemental material for Analysis of recurrent events in cluster randomised trials: The PLEASANT trial case study [file sj-docx-1-smm-10.1177_09622802251316972.docx]

# **Appendix**

# A PLEASANT Original Methodology

## A.1 Analysis Populations

Taking forward understanding of PLEASANT strengths, limitations and conclusions, it is sensible to focus the analysis of recurrent events on the primary (aged 5–-16 years), intention-to-treat population. This is due to difficulties in diagnosing asthma in children under 5 years old, given by Pedersen et al.^51^ and largely similar findings in the ITT and PP primary populations. This results in a primary population of 11564 participants (intervention group: 5631, control group: 5933), which includes 141 GP practices.

For the original PLEASANT analyses, GP practices with incomplete data for each relevant time period are excluded from analyses. Table 1.1) shows a summary of these sub populations, with these exclusions. Clearly, further into the study, there are more GP practice exclusions, resulting in at most 2474 participants (21.4% of the full primary population) removed from analysis populations. For clarity, data for the echo sub study time period of September 2014 is unavailable for this re-analysis, so does not feature in any subsequent analyses.

**Table A.1**: Summary of analysis sub populations (number of GP practices out of 141, or number of participants out of the 11564 primary population), excluding GP practices with incomplete data for each relevant time period

|  | Intervention | | Control |  | Total |  |
| --- | --- | --- | --- | --- | --- | --- |
| Time period | GP Practices | Participants | GP Practices | Participants | Participants |  |
| Unscheduled/ | |  |  |  |  |  |
| Total contacts | |  |  |  |  |  |
| Sep-13 | 68 | 5305 | 69 | 5586 | 10891 |  |
| Sep–Dec 13 | 65 | 5097 | 67 | 5384 | 10481 |  |
| Sep 13–Aug 14 | 58 | 4541 | 54 | 4549 | 9090 |  |
| Prescriptions | |  |  |  |  |  |
| Aug-13 | 68 | 5305 | 69 | 5586 | 10891 |  |
| Aug 13–Jul 14 | 58 | 4541 | 54 | 4549 | 9090 |  |
| Aug-14 | 58 | 4541 | 54 | 4549 | 9090 |  |

## A.2 Binary Model Methodology

In the PLEASANT study, a binary logistic regression model is used to analyse the proportion of participants with at least one unscheduled medical contact. This method is used to predict the odds of an unscheduled contact, i.e., a binary outcome (zero if no unscheduled contacts, one if one or more unscheduled contacts), based on the model covariates (participant age on 01-09-2013, gender, number of unscheduled contacts the previous September time period, treatment group (intervention, control)), including a random effect of GP practice to account for clustering (homogeneous groups of participants, by GP practice).

The natural logarithm of the odds, of an unscheduled medical contact, is a linear combination of the model covariates, which is calculated as a probability by the logistic function. This relationship is given by Menard^52^,

logit$(Y)=\ln\{\frac{P(Y=1)}{1-P(Y=1)}\}=\beta_{1}X_{1}+\beta_{2}X_{2}+...+\beta_{p}X_{p}$

where $p$ is number of covariates, $\beta_{1},\beta_{2},...,\beta_{p}$ are regression coefficients, often $X_{1}\equiv1$ to include an intercept

with probability mass function;

$$P(Y=1)=\frac{\exp(\beta_{1}X_{1}+\beta_{2}X_{2}+...+\beta_{p}X_{p})}{1+\exp(\beta_{1}X_{1}+\beta_{2}X_{2}+...+\beta_{p}X_{p})}=\frac{1}{1+\exp(-(\beta_{1}X_{1}+\beta_{2}X_{2}+...+\beta_{p}X_{p}))}$$

This method assumes a binary outcome variable, independent observations, a linear relationship between the logit of the outcome variable and model covariates, no multicollinearity between model covariates, and no extreme outliers.

The measure of group effect is usually the odds ratio (OR); essentially the ratio of the odds of an event in one group, divided by the odds of an event in the other group (holding all other variables fixed). This assesses the strength of association between the intervention and the outcome, giving a comparison of odds between groups. The same approach is used for total contacts and prescriptions.

## A.3 Binary Model Results

Analyses are performed using R package "lme4" (Bates et al.^53^) and "ResourceSelection" (Lele et al.^54^) for goodness of fit testing. Consistent with PLEASANT methods, analysis sub populations are used (excluding GP practices with incomplete data for each relevant time period, shown in Table A.1).

Table A.2 shows logistic model results for the primary outcome of the proportion of participants with an unscheduled contact in September 2013. There is no evidence to suggest a difference in odds of an unscheduled contact between groups (OR: 1.095, 95% Confidence Interval (CI): 0.961, 1.248, $p$=0.174). The odds ratio given for September 2013--August 2014 is in favour of the intervention group (reducing the odds of an unscheduled contact by 3.3%), however, is also statistically non-significant ($p$>0.05), as is September--December 2013.

**Table A.2**: Results of the proportion of participants with an unscheduled contact using binary (logistic regression) methods, for each time period (excluding GP practices with incomplete data for relevant time period).

|  | Intervention | Control |  | |
| --- | --- | --- | --- | --- |
| Time period | **Mean (SD)** | **Mean (SD)** | **Odds Ratio (95% CI)** | **p-value** |
| Sep-13 | 0.45 (0.50) | 0.44 (0.50) | 1.095 | 0.174 |
|  |  |  | (0.961, 1.248) | |
| Sep – Dec-13 | 0.88 (0.33) | 0.88 (0.33) | 1.099 | 0.188 |
|  |  |  | (0.955, 1.264) | |
| Sep-13 – Aug-14 | 0.99 (0.12) | 0.98 (0.14) | 0.967 | 0.702 |
|  |  |  | (0.815, 1.147) | |

Table A.3 shows equivalent results for total (scheduled, unscheduled) contacts, showing no evidence of an intervention effect, for any time period. The September 2013 and September 2013--August 2014 odds ratios are in favour of the intervention group (reducing odds of a total contact by 1.4% and 11%, respectively), however, are statistically non-significant ($p$>0.05).

**Table A.3**: Results of the proportion of participants with a total contact using binary (logistic regression) methods, for each time period (excluding GP practices with incomplete data for relevant time period).

|  | Intervention | Control |  | |
| --- | --- | --- | --- | --- |
| Time period | **Mean (SD)** | **Mean (SD)** | **Odds Ratio (95% CI)** | **p-value** |
| Sep-13 | 0.53 (0.50) | 0.53 (0.50) | 0.986 | 0.893 |
|  |  |  | (0.798, 1.217) | |
| Sep – Dec-13 | 0.92 (0.27) | 0.92 (0.27) | 1.059 | 0.530 |
|  |  |  | (0.885, 1.267) | |
| Sep-13 – Aug-14 | 0.99 (0.08) | 0.99 (0.09) | 0.890 | 0.312 |
|  |  |  | (0.711, 1.115) | |

Table A.4 shows logistic model results for prescriptions. There is strong evidence ($p$<0.001) to suggest the odds of collecting a prescription for the intervention group in August 2013, is 43.4% higher, compared to the control group (OR: 1.434, 95% CI: 1.247, 1.649), with all other variables held fixed. There is no evidence of a group difference for August 2014 or August 2013--July 2014. For August 2013–-July 2014, the binary model struggles to find accurate parameter estimates and confidence intervals (despite the Hosmer-Lemeshow test, given by Hosmer^55^, suggesting no evidence of poor fit), due to singularity issues (perfect correlation between variables), so an alternative model may be preferred.

**Table A.4**: Results of the proportion of participants with a steroid inhaler prescription using binary (logistic regression) methods, for each time period (excluding GP practices with incomplete data for relevant time period).

|  | Intervention | Control |  | |
| --- | --- | --- | --- | --- |
| Time period | **Mean (SD)** | **Mean (SD)** | **Odds Ratio (95% CI)** | **p-value** |
| Aug-13 | 0.16 (0.37) | 0.12 (0.33) | 1.434 | <0.001 |
|  |  |  | (1.247, 1.649) | |
| Aug-14 | 0.13 (0.33) | 0.12 (0.33) | 1.069 | 0.474 |
|  |  |  | (0.890, 1.285) | |
| Aug-13 – Jul-14 | 0.72 (0.45) | 0.71 (0.45) | 0.844 | 1.000 |
|  |  |  | (0, infinity) | |

Using the Hosmer Lemeshow test to assess goodness of fit for the logistic regression model, is reasonably satisfied for the remainder of the prescriptions time periods, as well as all time periods for unscheduled and total contacts.

## A.4 Negative Binomial Model Methodology

A negative binomial regression model, including the same model covariates (participant age on 01-09-2013, gender, number of unscheduled contacts the previous September time period, treatment group and random effect of GP practice), is used in the PLEASANT study to analyse number of unscheduled contacts.

This model determines the probability of a participant experiencing an unscheduled contact, adjusted by the given model covariates. This relationship is described by Hilbe^56^, using the probability mass function (pmf),

$$P(Y=y_{i}|\mu_{i},\delta)=\left( \frac{\Gamma(y_{i}+\frac{1}{\delta})}{\Gamma(y_{i}+1)\Gamma(\frac{1}{\delta})} \right)\left( \frac{1}{1+\delta\mu_{i}} \right)^{\frac{1}{\delta}}\left( 1-\frac{1}{1+\delta\mu_{i}} \right)^{y_{i}}$$

where $\mu_{i}$ is the mean incidence rate of $y_{i}=\exp(\beta_{1}X_{1i}+\beta_{2}X_{2i}+...+\beta_{p}X_{pi})$, for observation $i$, often $X_{1}\equiv1$ to include an intercept, $\delta$ is the overdispersion parameter, with all remaining notation as previously defined.

The model assumes linearity in model covariates, independent observations and a larger conditional variance than conditional mean. Incidence rate ratios (IRR) can be used; essentially a ratio of two group means (incidence rates), with all other variables held fixed, often expressed as a percentage point change for ease of interpretation. The same approach is used for total contacts and prescriptions.

## A.5 Negative Binomial Model Results

Analyses are performed using R package "lme4" (Bates et al.^53^), excluding GP practices with incomplete data for each relevant time period. Table 1.5 shows the negative binomial model results for unscheduled contacts. There is no evidence to suggest a group difference in the number of unscheduled contacts, in September 2013 (IRR: 1.021, 95% CI: 0.935, 1.115, $p$=0.639). Both incidence rate ratios for September--December 2013 and September 2013--August 2014 are in favour of the intervention group (reducing unscheduled contacts by 2.0% and 2.7%, respectively), however are statistically non-significant.

**Table A.5**: Negative binomial model results of the number of unscheduled contacts (excluding GP practices with incomplete data for the relevant time period).

|  | Intervention | Control |  | |
| --- | --- | --- | --- | --- |
| Time period | **Mean (SD)** | **Mean (SD)** | **Rate Ratio**  **(95% CI)** | **p-value** |
| Sep-13 | 0.81 (1.24) | 0.81 (1.32) | 1.021 | 0.639 |
|  |  |  | (0.935, 1.115) | |
| Sep – Dec-13 | 3.19 (3.46) | 3.32 (3.78) | 0.980 | 0.504 |
|  |  |  | (0.925, 1.039) | |
| Sep-13 – Aug-14 | 9.08 (8.44) | 9.37 (9.34) | 0.973 | 0.220 |
|  |  |  | (0.932, 1.016) | |

Table A.6 shows equivalent results for total contacts. There is statistically significant, moderate evidence ($p$=0.025) of a group difference (all other variables held fixed), suggesting a 5.1% decrease in total contacts for the intervention group, compared to the control group, in September 2013--August 2014 (IRR:0.949, 95% CI: 0.907, 0.994). There is no evidence of a group difference for remaining time periods, however, both statistically non-significant rate ratios are in favour of the intervention group (Sep 2013 IRR: 0.966, Sep--Dec 2013 IRR: 0.955).

**Table A.6**: Negative binomial results of the number of total contacts (excluding GP practices with incomplete data for the relevant time period).

|  | Intervention | Control |  | |
| --- | --- | --- | --- | --- |
| Time period | **Mean (SD)** | **Mean (SD)** | **Rate Ratio**  **(95% CI)** | **p-value** |
| Sep-13 | 1.05 (1.44) | 1.10 (1.58) | 0.966 | 0.525 |
|  |  |  | (0.868, 1.075) | |
| Sep – Dec-13 | 4.31 (4.22) | 4.53 (4.52) | 0.955 | 0.148 |
|  |  |  | (0.897, 1.017) | |
| Sep-13 – Aug-14 | 11.52 (10.15) | 12.08 (11.17) | 0.949 | 0.025 |
|  |  |  | (0.907, 0.994) | |

Table A.7 shows negative binomial model results for number of prescriptions, in August 2013, August 2014 and August 2013-–July 2014. There is strong evidence (p<0.001) to suggest the intervention group have 1.323 (95% CI: 1.176, 1.489) times the rate of prescriptions in August 2013 compared to the control group, equivalent to a 32.3% increase (supporting this aim of the trial). There is also strong evidence ($p$=0.007) that the intervention group have a 7.4% increase in prescriptions (HR:1.074, 95% CI: 1.019, 1.132) in August 2013-–July 2014, compared to the control group. The August 2014 rate ratio is in favour of the intervention group, suggesting a statistically non-significant 1.5% increase.

**Table A.7**: Negative binomial results of the number of steroid inhaler prescriptions (excluding GP practices with incomplete data for the relevant time period).

|  | Intervention | Control |  | |
| --- | --- | --- | --- | --- |
| Time period | **Mean (SD)** | **Mean (SD)** | **Rate Ratio**  **(95% CI)** | **p-value** |
| Aug-13 | 0.17 (0.40) | 0.13 (0.36) | 1.323 | <0.001 |
|  |  |  | (1.176, 1.489) | |
| Aug-14 | 0.14 (0.36) | 0.13 (0.37) | 1.015 | 0.854 |
|  |  |  | (0.863, 1.195) | |
| Aug-13 – Jul-14 | 2.20 (2.81) | 2.09 (2.84) | 1.074 | 0.007 |
|  |  |  | (1.019, 1.132) | |

Checking the negative binomial model assumption of the conditional mean not equal to the conditional variance, the likelihood ratio test showed that the negative binomial model is more appropriate than the Poisson model for unscheduled contacts, total contacts and prescriptions, for each time period.

## A.6 Cox Proportional Hazards Methodology

PLEASANT study uses a Cox proportional hazards model with shared frailty, including the aforementioned model covariates (participant age on 01-09-2013, gender, number of contacts the previous September time period, treatment group (intervention, control)). The Cox proportional hazards model analyses time to first unscheduled medical contact, so includes all GP practices (whether complete or incomplete data for the specified time period). The shared frailty part of the model are random effects to account for clustering by GP practices. Similarity of participants within a GP practice (cluster) is accounted for, by sharing the same frailty (random effect) within the cluster. As discussed by Balan and Putter^29^, the frailty (often assumed to follow a gamma distribution for ease of interpretation) essentially has a multiplicative effect on the hazard function, shown as follows,

$$\lambda_{ig}(t|\alpha_{g})=\alpha_{g}\lambda_{ig}(t)=\alpha_{g}\lambda_{0}(t)\exp(X_{i}\beta^{T})$$

                          $=\alpha_{g}\lambda_{0}(t)\exp(\beta_{1}X_{1i}+\beta_{2}X_{2i}+...+\beta_{p}X_{pi})$

for participant $i$ in cluster $g$, with N clusters (GP practices), where $n_{g}$ participants are in cluster $g$, sharing frailty term $\alpha_{g}$, with all remaining notation as previously defined.

This represents a participants hazard rate; the risk (probability) of an unscheduled contact, at particular time points during the analysis time periods, based on the baseline hazard function, model covariates and cluster random effect. For analysis, participants with zero events are censored, with GP practice follow-up time taken into account within survival time.

The main assumption for the Cox model is proportional hazards, where the model covariates are assumed to have a multiplicative relationship to the hazard, that remains constant over time. This means the hazard ratio (HR: ratio of hazard rates between groups, representing instantaneous risk during the time period) does not vary over time.

The same approach is used for total (unscheduled and scheduled) contacts. For clarity, time to first prescription was not an outcome of the PLEASANT trial, so analysis of prescriptions using this model type are not carried out.

## A.7 Cox Proportional Hazards Results

Cox Proportional Hazards (shared frailty) analyses are performed using R packages "survival" (Therneau and Grambsch^38^, Therneau^39^), "coxme" (Therneau^57^) and "survminer" (Kassambara et al.^58^) for Kaplan Meier plots.

Figure A.1 shows a Kaplan Meier plot for time (in days from 01-09-2013) to first unscheduled contact during September 2013, for the primary population. This plots the ‘survival’ function (probability of a participant not experiencing an event during the time period), for each group. At risk of event numbers are shown at various time points (those that have experienced an event, or censored are removed). There are similar group survival curves and ‘at risk’ numbers, suggesting no evidence of a group difference for time to first unscheduled contact.


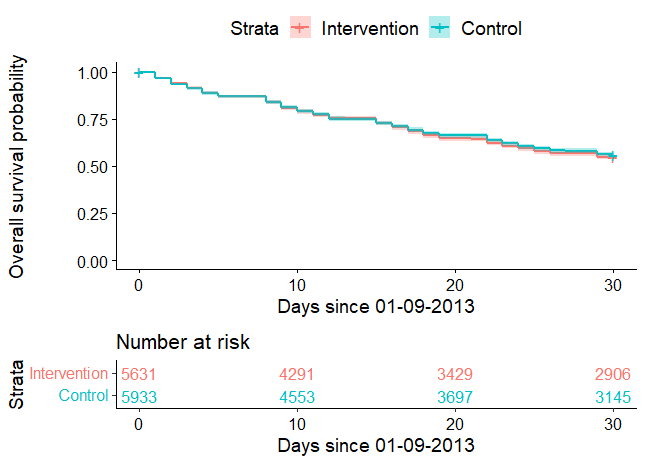


**Figure A.1**: Kaplan Meier plot; time(days) to first unscheduled contact in Sep-13.

Kaplan Meier plots for unscheduled contacts for September–-December 2013 and September 2013–-August 2014 are shown as follows, in Figures A.2 and A.3, with no difference evident between groups.


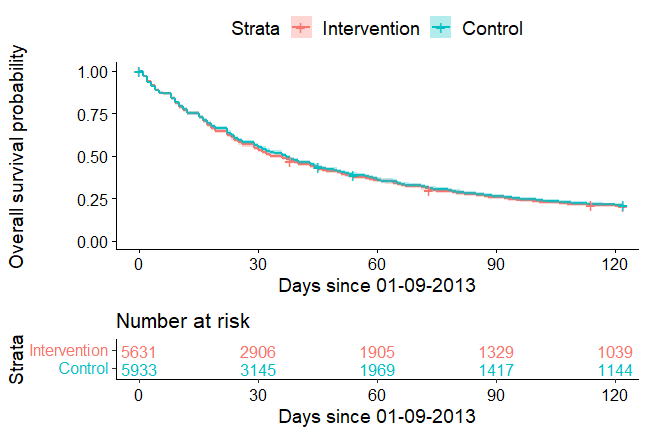


**Figure A.2**: Kaplan Meier plot of time (days) to first unscheduled contact in Sep--Dec-2013.


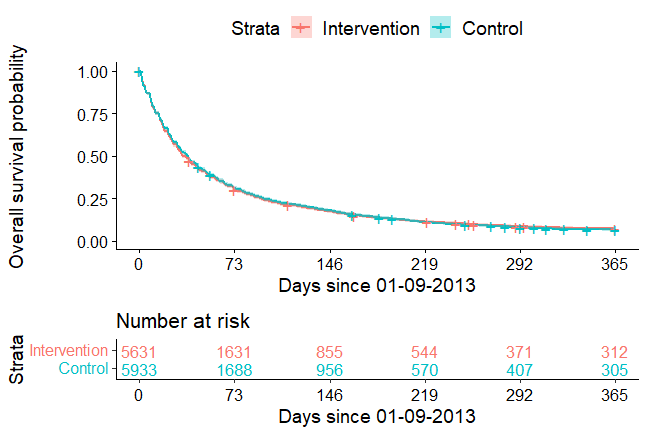


**Figure A.3**: Kaplan Meier plot of time (days) to first unscheduled contact in Sep-13--Aug-14.

Table A.8 shows Cox shared frailty results for unscheduled contacts. There is no evidence to suggest the intervention group, at any particular time point in September 2013, are more or less likely to have an unscheduled contact than the control group (HR: 1.059, 95% CI 0.966, 1.162). There is also no evidence of a group difference in risk for September--December 2013 or September 2013–-August 2014, both also in favour of the control group.

**Table A.8**: Cox shared frailty results of time (days) to first unscheduled contact.

|  | Intervention | Control |  | |
| --- | --- | --- | --- | --- |
| Time period | **Median (days)** | **Median (days)** | **Hazard Ratio**  **(95% CI)** | **p-value** |
| Sep-13 | 11 | 11 | 1.059 | 0.220 |
|  |  |  | (0.966, 1.162) | |
| Sep – Dec-13 | 24 | 24 | 1.058 | 0.110 |
|  |  |  | (0.988, 1.133) | |
| Sep-13 – Aug-14 | 30 | 32 | 1.043 | 0.220 |
|  |  |  | (0.975, 1.116) | |

Kaplan-Meier plots for total contacts (results not shown) are found to be similar to those for unscheduled contacts, with survival curves and ‘at risk’ numbers similar by group. Table A.9 shows Cox shared frailty results, with no evidence of a group difference in risk of total contacts, for any time period. All hazard ratios are in favour of the intervention group (reducing risk of total contacts by 0.3%-2.2%), but are statistically non-significant ($p$>0.05).

**Table A.9**: Cox shared frailty model results of time (days) to first total contact.

|  | Intervention | Control |  | |
| --- | --- | --- | --- | --- |
| Time period | **Median (days)** | **Median (days)** | **Hazard Ratio**  **(95% CI)** | **p-value** |
| Sep-13 | 11 | 10 | 0.978 | 0.770 |
|  |  |  | (0.844, 1.134) | |
| Sep - Dec-13 | 22 | 19 | 0.997 | 0.960 |
|  |  |  | (0.890, 1.118) | |
| Sep-13 – Aug-14 | 24 | 24 | 0.992 | 0.890 |
|  |  |  | (0.889, 1.107) | |

Assessing the proportional hazards assumption, using Schoenfeld residuals, offered by Therneau and Grambsch^38^, appears reasonably satisfied for unscheduled contacts and total contacts, for each time period.

# B Conditional Frailty Model

## B.1 Data Structure

The following table (Table B.1) shows the data structure required in R (version 4.0.5) statistical software (R Core Team^59^) for the conditional frailty model (using fictitious data). To clarify, the start and stop times are the gap times between events, count is the cumulative event count per participant and status is whether the participant is censored (0) or not (1). Here, participant (ID) 1 has 2 events, then is censored at the end of their follow up time (when their GP practice (GP.ID) stops providing data) of 20 days from the start of the time period. Participant 2 has a follow up time of 29 days from the start of the time period and has zero events, so is censored. Participant 3 experiences 1 event and completed the full time period.

**Table B.1**: Data structure in R for the Conditional frailty model (fictitious data).

| ID | Group | Age | Gender | GP.ID | Event.date | Start | Stop | Count | Status |
| --- | --- | --- | --- | --- | --- | --- | --- | --- | --- |
| 1 | Intervention | 8.1 | Male | 10 | ######## | 0 | 10 | 1 | 1 |
| 1 | Intervention | 8.1 | Male | 10 | ######## | 10 | 11 | 2 | 1 |
| 1 | Intervention | 8.1 | Male | 10 | N/A | 11 | 20 | 3 | 0 |
| 2 | Control | 10.2 | Female | 21 | N/A | 0 | 29 | 1 | 0 |
| 3 | Control | 12.3 | Male | 34 | ######## | 0 | 19 | 1 | 1 |

## B.2 R Code for a Global Estimate

The conditional frailty model code using R package "survival" (Therneau and Grambsch^38^, Therneau^39^) involves using the data structure in Appendix B1 Table B.1. The model uses the ‘survival time’ between each event as 'Start – Stop' times (gap times), cumulative event counts ('Count') per participant for the strata (to account for event dependence) and a frailty term for the GP practices (GP.ID) with a gamma distribution (to account for clustering), plus the remaining model covariates.

library(survival)
cf_mod <- coxph(Surv(Stop-Start, status) ~ event_count_prev_year
 + group + gender + age + frailty(GP.ID,
 distribution = "gamma")
 + strata(Count), data = data)

## B.3 R Code for Event-Specific Results

This code builds upon the conditional frailty model code using R package "survival" (Therneau and Grambsch^38^, Therneau^39^) in Appendix B2. To obtain event-specific intensity ratios, that depend upon the strata (event number, each with a different baseline hazard) and the treatment group, the model forumla is re-arranged in the following way. This model forumla re-arrangement is used and supported by Abreu and Sousa-Ferreira^42^ for event-specific results.

library(survival)
cf_mod_strata <- coxph(Surv(Stop-Start, status) ~ strata(Count)
 / (group + event_count_prev_year + gender
 + age) + frailty(GP.ID, distribution =
 "gamma"), data = data)

## B.4 R Code for a Rare Events Bias Adjustment

Based on the model structure used in Appendix B1 Table B.1, to set up the conditional frailty model including Firth's rare events adjustment involves using R package "coxphf" (Heinze et al.^40^) to construct the model, then adding the 'firth=TRUE' statement to the model code.

library(coxphf)
cf_firth <- coxphf(Surv(Stop-Start, status) ~ event_count_prev_year
 + group + gender + age + frailty(GP.ID,
 distribution = "gamma") + strata(Count),
 data = data, firth = TRUE)

# C Total Contacts Diagnostics

Testing event dependence within the conditional frailty model, using cumulative hazard plots (Figures C.1 - C.3), show that baseline hazards vary by event number, with increasing risk of subsequent events, for each time period. This satisfies the assumption of event dependence, justifying the structure of event stratification within the model.


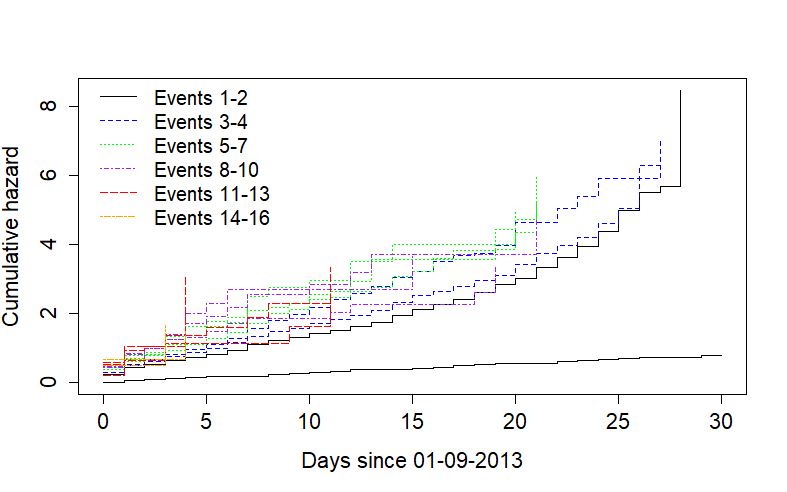


**Figure C.1**: Cumulative hazard plot by event number for the conditional frailty model, for total contacts, over September 2013.


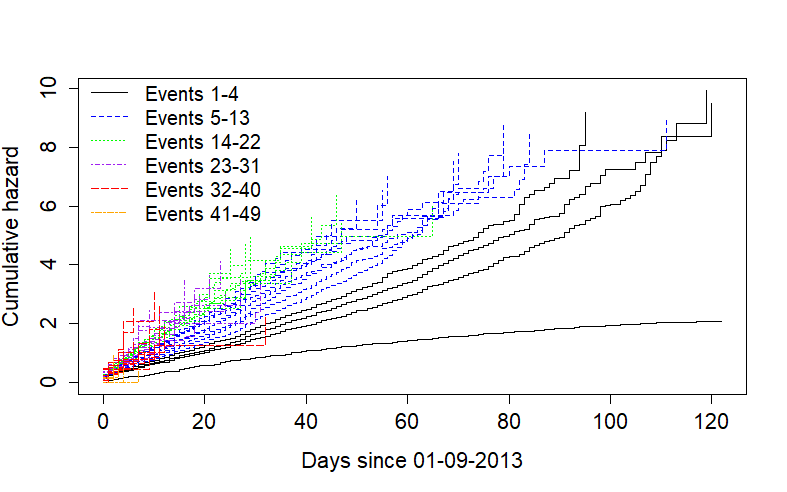


**Figure C.2**: Cumulative hazard plot by event number for the conditional frailty model, for total contacts, over September - December 2013.


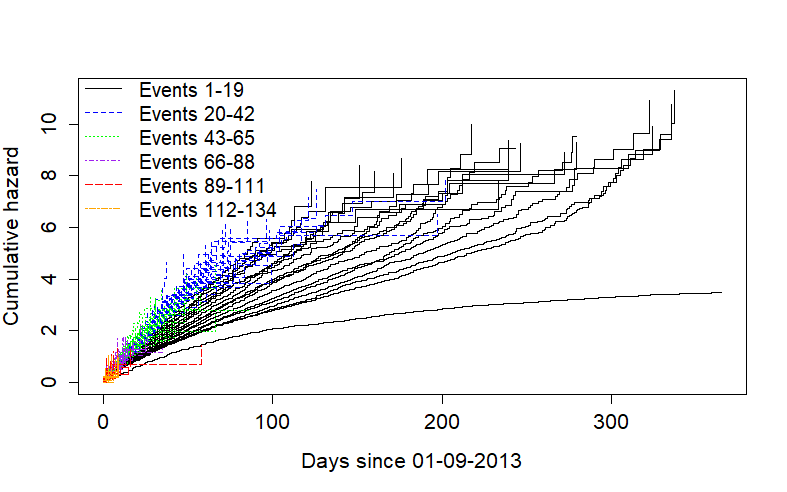


**Figure C.3**: Cumulative hazard plot by event number for the conditional frailty model, for total contacts, in September 2013 - August 2014.

# D Total Contacts Sensitivity Analysis

## D.1 Truncation Points

Table D.1 shows the cap of maximum number of events per participant at each truncation point (includes event risk sets that contain above a particular percentage of the population; 5%, 2%, 1%, 0.5%, 0.25%), for each time period. This ranges from 4-10 events for September 2013, 12-30 events for September--December 2013 and 30-67 events for September 2013--August 2014. Without truncation, there is a maximum number of events per participant of 16, 49 and 134, for time periods September 2013, September--December 2013 and September 2013--August 2014, respectively. Hence, many of the later risk sets have been discarded, especially over the full year.

**Table D.1**: Maximum number of total contacts per participant for each truncation dataset (includes event risk sets that contain above the specified percentage of the population), at each time period.

| Time Period | No truncation | >5% | >2% | >1% | >0.5% | >0.25% |
| --- | --- | --- | --- | --- | --- | --- |
| Sep-13 | 16 | 4 | 5 | 7 | 8 | 10 |
| Sep – Dec-13 | 49 | 12 | 16 | 20 | 24 | 30 |
| Sep-13 – Aug-14 | 134 | 30 | 40 | 49 | 58 | 67 |

When testing for event dependence (using cumulative hazard plots) and clustering (using the likelihood ratio test), for the truncated datasets, the diagnostics suggest that the conditional frailty model is appropriate (results not given as these are very similar to the full event risk set results).

## D.2 Truncated Conditional Frailty Model Results

Table D.2 shows the conditional frailty model results for the truncated datasets, for total contacts, for each time period. For September 2013, the truncated results differ marginally to the full primary data results when using truncation points >5% (max 4 events) and >2% (max 5 events), with a 0.007 larger intensity ratio (IR) of 0.996 (closer to 1) and a 0.07 wider confidence interval. When using truncation points >1%, >0.5% and >0.25%, the results are very consistent to when using all event risk sets, in terms of size, direction of effect and uncertainty (confidence interval width). For time periods September--December 2013 and September 2013--August 2014, all the truncation results are again largely consistent with the results using all event risk sets.

Interestingly, for truncation point >2% (max 40 events) during September 2013--August 2014, $p$=0.098. This may suggest very weak evidence of a 2.5% risk reduction in total contacts for the intervention group, compared to the control group, at a particular time point during September 2013--August 2014. However, all other truncation points consistently give $p$>0.1. Furthermore, all results remain statistically non-significant ($p$>0.05).

**Table D.2**: Conditional frailty model results for group allocation (total contacts), for the truncated datasets.

| Time Period | >5% | >2% | >1% | >0.5% | >0.25% |  |
| --- | --- | --- | --- | --- | --- | --- |
| Sep-13 |  |  |  |  |  |  |
| IR | 0.996 | 0.996 | 0.987 | 0.988 | 0.988 |  |
| 95% CI | (0.923,1.075) | (0.923,1.074) | (0.917,1.064) | (0.918,1.064) | (0.918,1.063) | |
| p-value | p = 0.925 | p = 0.907 | p = 0.739 | p = 0.757 | p = 0.743 |  |
| Sep - Dec-13 | |  |  |  |  |  |
| IR | 0.970 | 0.970 | 0.971 | 0.971 | 0.971 |  |
| 95% CI | (0.929,1.012) | (0.930,1.012) | (0.931,1.013) | (0.931,1.013) | (0.931,1.013) | |
| p-value | p = 0.162 | p = 0.164 | p = 0.170 | p = 0.172 | p = 0.174 |  |
| Sep-13 - Aug-14 | |  |  |  |  |  |
| IR | 0.976 | 0.975 | 0.976 | 0.977 | 0.977 |  |
| 95% CI | (0.947,1.006) | (0.946,1.005) | (0.947,1.005) | (0.948,1.006) | (0.948,1.007) | |
| p-value | p = 0.112 | p = 0.098 | p = 0.107 | p = 0.122 | p = 0.129 |  |

## D.3 Using a Log-Normal Distribution for the Frailty Term

Table D.3 shows statistically significant results in the LRT and Wald test for the frailty term when using a log-Normal distribution. In line with the frailty term gamma distribution results, this gives justification for the presence of clustering within these data. The frailty term variance is marginally lower (slightly lower level of heterogeneity between GP practices) when using a log-Normal distribution, compared to when using a gamma distribution, but relatively similarly low.

**Table D.3**: Frailty term (total contacts conditional frailty model) results, using a log-Normal distribution, for each time period, using the likelihood ratio test (LRT), Wald test and variance.

| Time period | LRT p-value | Wald test p-value | Variance |
| --- | --- | --- | --- |
| Sep-13 | <0.001 | <0.001 | 0.0224 |
| Sep – Dec-13 | <0.001 | <0.001 | 0.0099 |
| Sep-13 – Aug-14 | <0.001 | <0.001 | 0.0047 |

Results for the CF model using a log-Normal distribution for the frailty term, for total medical contacts, are given in Table D.4, providing global estimates of the effect. The intensity ratios, confidence interval width and statistical significance, are all consistent to the CF model results and conclusions when using a gamma distribution for the frailty term.

**Table D.4**: Conditional frailty model results (using a log-Normal distribution for the frailty term) for group allocation (total contacts).

| Time period | Intensity Ratio (IR) | 95% CI | p-value |
| --- | --- | --- | --- |
| Sep-13 | 0.993 | (0.930, 1.060) | 0.829 |
| Sep – Dec-13 | 0.968 | (0.930, 1.007) | 0.110 |
| Sep-13 – Aug-14 | 0.978 | (0.951, 1.005) | 0.106 |

# E Unscheduled Contacts Analysis

Table E.1 gives the proportion of participants with a maximum of zero, one, or more than one (multiple) unscheduled event, for each time period. There are a fairly large number (and proportion) of participants with recurrent events (multiple events: 2094 (18.1%) in September 2013, 6716 (58.1%) in September--December 2013, 9212 (79.7%) in September 2013--August 2014). Therefore, penalty adjustments for rare events bias are not justified for these data for unscheduled contacts.

**Table E.1**: Number and proportion of participants with a maximum of zero, one or multiple unscheduled events for each time period, out of 11564 participants.

|  | Maximum per participant | | | Total Events |
| --- | --- | --- | --- | --- |
| Time period | **0 events** | **1 event** | **Multiple events** |  |
| Sep-13 | 6724 (58.1%) | 2746 (23.7%) | 2094 (18.1%) | 8814 |
| Sep – Dec-13 | 2988 (25.8%) | 1860 (16.1%) | 6716 (58.1%) | 34668 |
| Sep-13 – Aug-14 | 1603 (13.9%) | 749 (6.5%) | 9212 (79.7%) | 93825 |

Testing for clustering, Table E.2 gives results of the likelihood ratio test (and Wald test) to assess the statistical significance of the frailty term, for unscheduled contacts. A statistically significant result ($p<0.001$), suggests strong evidence to include the model frailty term, for each time period, to better describe these data. The frailty term variance is small at 0.0109, 0.0114 and 0.0056 for September 2013, September--December 2013 and September 2013--August 2014, respectively, perhaps suggesting a low level of heterogeneity between GP practices.

**Table E.2**: Frailty term (unscheduled events conditional frailty model) results for each time period, using the likelihood ratio test (LRT), Wald test and variance.

| Time period | LRT p-value | Wald test p-value | Variance |
| --- | --- | --- | --- |
| Sep-13 | <0.001 | <0.001 | 0.0109 |
| Sep – Dec-13 | <0.001 | <0.001 | 0.0114 |
| Sep-13 – Aug-14 | <0.001 | <0.001 | 0.0056 |

Cumulative hazard plots (Figures E.1 - E.3) for the conditional frailty model show baseline hazards vary by unscheduled event, for September 2013, September--December 2013 and September 2013--August 2014, respectively, justifying event dependence. Time to an unscheduled event is shorter as the event number increases, showing an increasing risk of subsequent events.


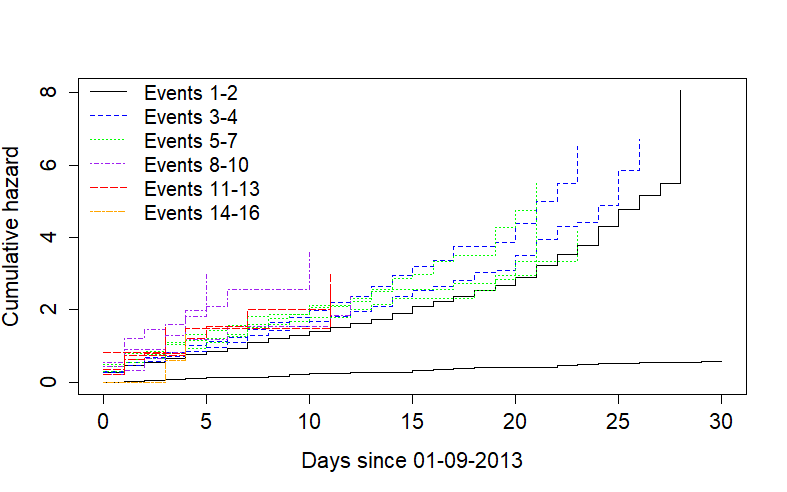


**Figure E.1**: Cumulative hazard plot by event number for the conditional frailty model, for unscheduled contacts, over September 2013.


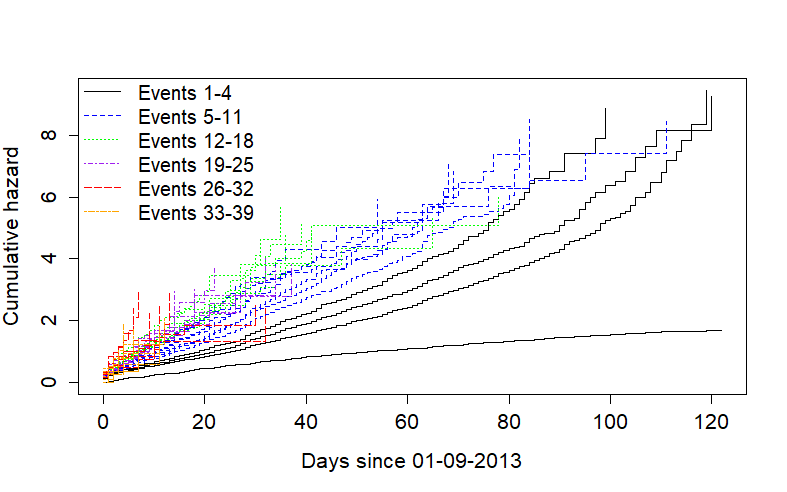


**Figure E.2**: Cumulative hazard plot by event number for the conditional frailty model, for unscheduled contacts, over September - December 2013.


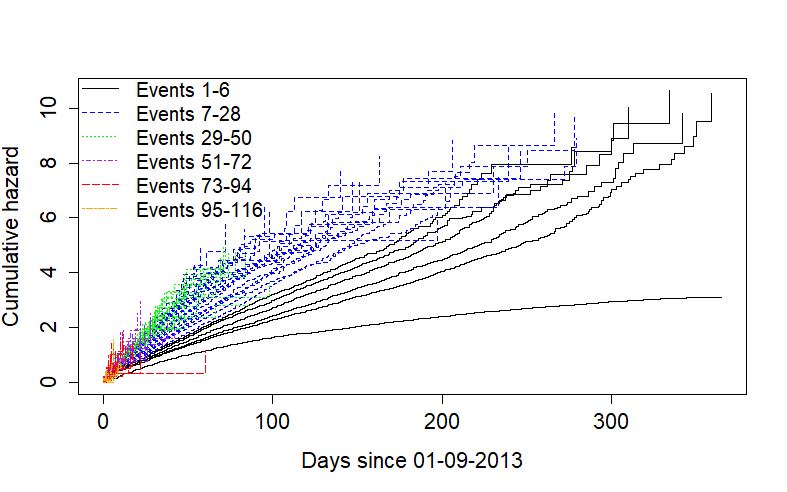


**Figure E.3**: Cumulative hazard plot by event number for the conditional frailty model, for unscheduled contacts, over September 2013 - August 2014.

Results for the conditional frailty model, for unscheduled contacts, are given in Table E.3. The intensity ratios for September--December 2013 and September 2013--August 2014 are in favour of the intervention group (2.6%, 1.6% risk reduction of an unscheduled contact, respectively, compared to the control group), but in favour of the control group for September 2013 (2.4% risk increase for the intervention group). However, these results are statistically non-significant ($p$>0.05), for each of the time periods September 2013 (IR:1.024, 95% CI: 0.967, 1.084, $p$=0.419), September--December 2013 (IR:0.974, 95% CI: 0.932, 1.017, $p$=0.234), or September 2013--August 2014 (IR:0.984, 95% CI: 0.955, 1.014, $p$=0.287).

**Table E.3**: Conditional frailty model results for group allocation (unscheduled).

| Time period | Intensity Ratio (IR) | 95% Confidence Interval | p-value |
| --- | --- | --- | --- |
| Sep-13 | 1.024 | (0.967, 1.084) | 0.419 |
| Sep – Dec-13 | 0.974 | (0.932, 1.017) | 0.234 |
| Sep-13 – Aug-14 | 0.984 | (0.955, 1.014) | 0.287 |

# F Unscheduled Contacts Sensitivity Analysis

## F.1 Truncation Points

As previously discussed, the conditional frailty model risk sets for later events can become too small for reliable estimates, with a solution of a using truncated datasets to exclude these risk sets. Table F.1 shows the maximum number of unscheduled events per participant, for each truncated dataset (includes event risk sets that contain above a particular percentage of the population; 5%, 2%, 1%, 0.5%, 0.25%), for each time period. There are a maximum of 3-8 events for September 2013, 10-25 events for September--December 2013 and 24-55 events for September 2013--August 2014. With no truncation, there is a maximum number of events of 16, 39 and 116 for time periods September 2013, September--December 2013 and September 2013--August 2014 respectively. Hence, this excludes many of the later events, particularly over the full year time period.

**Table F.1**: Maximum number of unscheduled events per participant for each truncation dataset (includes event risk sets that contain above the specified percentage of the population), at each time period.

| Time period | No truncation | >5% | >2% | >1% | >0.5% | >0.25% |
| --- | --- | --- | --- | --- | --- | --- |
| Sep-13 | 16 | 3 | 4 | 6 | 7 | 8 |
| Sep – Dec-13 | 39 | 10 | 13 | 17 | 20 | 25 |
| Sep-13 – Aug-14 | 116 | 24 | 33 | 41 | 48 | 55 |

Tests for event dependence (using cumulative hazard plots) and clustering (using the LRT for the frailty term), for the truncated datasets at each time period, show that the conditional frailty model is appropriate in each case (results not provided as these are very similar to using the full event risk sets).

## F.2 Truncated Conditional Frailty Model Results

Table F.2 shows the conditional frailty model results, for unscheduled events, using the truncated datasets for each time period.

The results for the September 2013 truncated datasets differ marginally to the full primary data results when removing the most event risk sets (including risk sets >5% (max 3 events), >2% (max 4 events) of the primary population). Compared to using all event risk sets, the intensity ratio (IR) is 0.011 larger (truncated IR: 1.035 compared to full risk sets IR: 1.024) in the same direction, with the 95% confidence intervals similarly sized. Results for truncation points >1%, >0.5%, >0.25% are very similar to when including all event risk sets. Overall, results are consistent in direction, size of effect and uncertainty (confidence interval width) to using all event risk sets.

For September--December 2013, when using truncation point >5% (max 10 events), the intensity ratio is marginally larger by 0.003 in the same direction, at 0.977. All other truncation point results are also very consistent in size, direction of effect and uncertainty, to using all event risk sets. For September 2013--August 2014, the intensity ratios and confidence interval width results again remain largely consistent, regardless of the population event risk sets used.

For all time periods and truncation points, the statistically non-significant result remains the same ($p$>0.05), suggesting stability in the conclusion of no evidence of a group difference in the risk of an unscheduled contact.

**Table F.2**: Conditional frailty model results for group allocation (unscheduled events), for the truncated datasets.

| Time period | >5% | >2% | >1% | >0.5% | >0.25% |  |
| --- | --- | --- | --- | --- | --- | --- |
| Sep-13 |  |  |  |  |  |  |
| IR | 1.035 | 1.035 | 1.024 | 1.024 | 1.025 |  |
| 95% CI | (0.973,1.102) | (0.975,1.099) | (0.966,1.086) | (0.966,1.086) | (0.967,1.087) | |
| p-value | p = 0.276 | p = 0.260 | p = 0.417 | p = 0.418 | p = 0.399 |  |
| Sep - Dec-13 | |  |  |  |  |  |
| IR | 0.977 | 0.974 | 0.974 | 0.974 | 0.974 |  |
| 95% CI | (0.936,1.021) | (0.932,1.017) | (0.932,1.017) | (0.932,1.017) | (0.933,1.018) | |
| p-value | p = 0.298 | p = 0.227 | p = 0.235 | p = 0.228 | p = 0.244 |  |
| Sep-13 - Aug-14 | |  |  |  |  |  |
| IR | 0.982 | 0.982 | 0.983 | 0.983 | 0.983 |  |
| 95% CI | (0.954,1.011) | (0.954,1.011) | (0.955,1.012) | (0.955,1.012) | (0.955,1.013) | |
| p-value | p = 0.224 | p = 0.219 | p = 0.241 | p = 0.259 | p = 0.261 |  |

# G Prescriptions Analysis

Table G.1 gives the proportion of participants with maximum counts of (steroid inhaler) prescriptions, for each time period. There appears to be very low counts (and proportions) of participants with recurrent prescriptions during August 2013 and August 2014, but a reasonable amount for August 2013--July 2014 (102 (0.9%), 78 (0.7%), 4544 (39.3%), respectively). Therefore, it appears a rare events adjustment is not required for August 2013--July 2014.

**Table G.1**: Number and proportion of participants with a maximum of zero, one and multiple prescriptions, for each time period, out of 11564 participants.

|  | Maximum per participant | | | Total Events |
| --- | --- | --- | --- | --- |
| Time period | **0 events** | **1 event** | **Multiple events** |  |
| Aug-13 | 9979 (86.3%) | 1483 (12.8%) | 102 (0.9%) | 1691 |
| Aug-14 | 10427 (90.2%) | 1059 (9.2%) | 78 (0.7%) | 1220 |
| Aug-13 – Jul-14 | 5145 (44.5%) | 1875 (16.2%) | 4544 (39.3%) | 21838 |

However, Firth’s penalised likelihood is applied for time periods August 2013 and August 2014, due to the low number of participants experiencing recurrent events. Conditional frailty model results are provided with and without the rare events adjustment, for comparison.

Example R code using Firth's rare events adjustment is given in Appendix B3.

Cumulative hazard plots for prescriptions, for each time period (Figures G.1 - G.3) show baseline hazards vary by event number, with increasing risk of subsequent events, justifying event strata for prescriptions data.


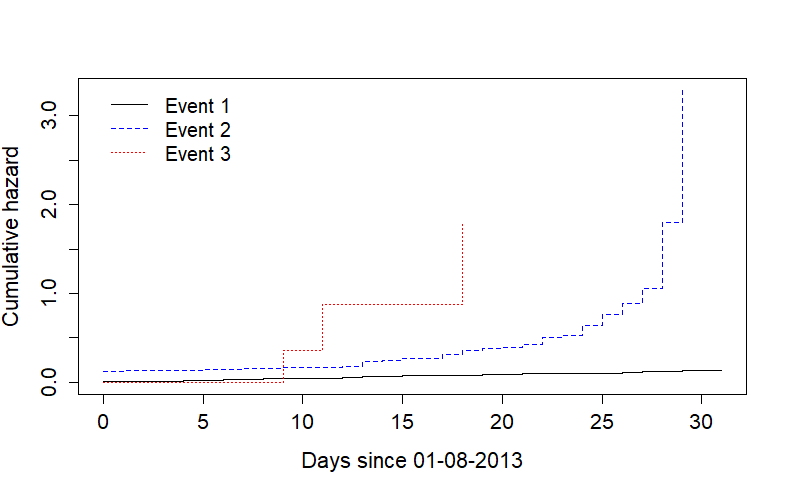


**Figure G.1**: Cumulative hazard plot by event number for the conditional frailty model, for prescriptions, over August 2013.


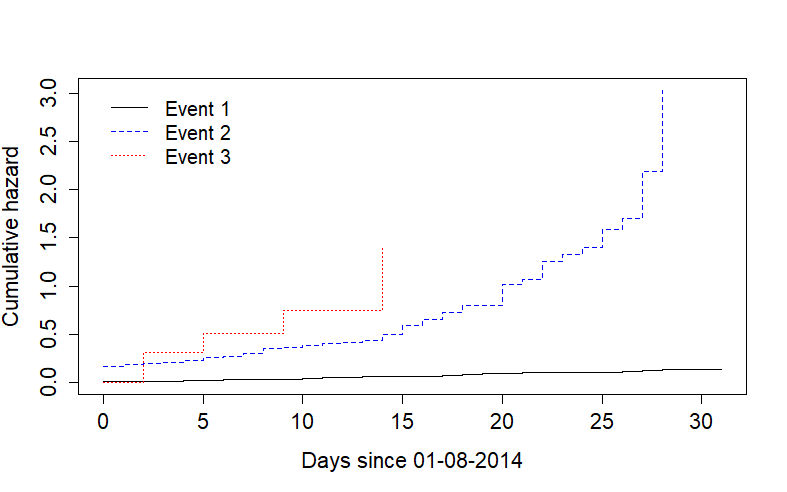


**Figure G.2**: Cumulative hazard plot by event number for the conditional frailty model, for prescriptions, over August 2014.


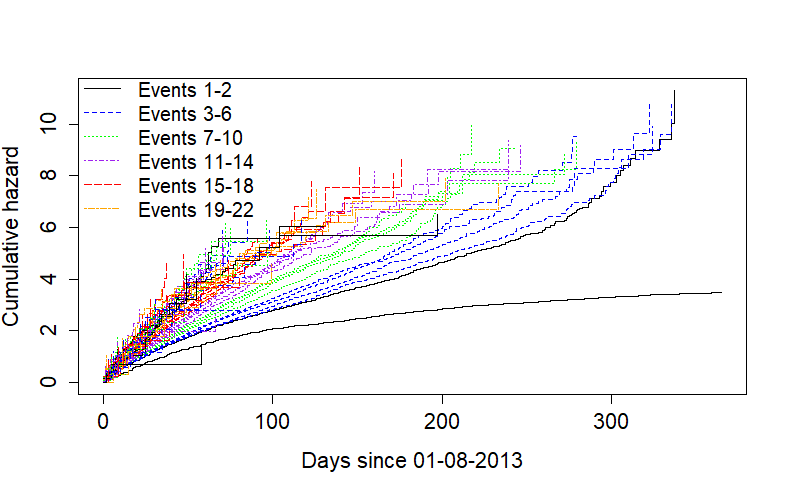


**Figure G.3**: Cumulative hazard plot by event number for the conditional frailty model, for prescriptions, in August 2013 - July 2014.

Table G.2 shows statistically significant likelihood ratio test results for the frailty term, satisfying the clustering assumption. Similar to unscheduled and total contacts, the prescriptions frailty term variance is low (Aug 2013: 0.0585, Aug 2014: 0.0834, Aug 2013--Jul 2014: 0.0166).

**Table G.2**: Frailty term (prescriptions conditional frailty model) results for each time period, using the likelihood ratio test (LRT), Wald test and variance.

| Time period | LRT p-value | Wald test p-value | Variance |
| --- | --- | --- | --- |
| Aug-13 | <0.001 | <0.001 | 0.0585 |
| Aug-14 | <0.001 | <0.001 | 0.0834 |
| Aug-13 – Jul-14 | <0.001 | <0.001 | 0.0166 |

Table G.3 shows prescriptions conditional frailty model results. There is strong evidence to suggest a 33.1% increased risk of a prescription (collection) in the intervention group (IR:1.331, 95% CI: 1.167, 2.259, $p$<0.001), compared to the control group, at any particular point in August 2013. The confidence interval for the intensity ratio is wide, suggesting lack of precision, but does not include 1. Table 7.4 shows equivalent results using Firth’s adjustment, remaining statistically significant for August 2013, with a slightly lower intensity ratio (IR:1.295, 95% CI: 1.176, 1.427, $p$<0.001). This suggests the intervention group experience a 29.5% risk increase of a prescription (collection), at any particular point in August 2013, compared to the control group. The confidence interval is narrower (by 0.841) using the rare events adjustment, suggesting better accuracy.

For August 2014, the rare events adjustment results show a 0.011, marginally smaller intensity ratio and 0.102 narrower confidence interval (IR: 1.018, 95% CI: 0.909, 1.141, $p$=0.752), compared to without the adjustment (IR: 1.029, 95% CI: 0.875, 1.209, $p$=0.732). These adjusted results show a statistically non-significant 1.8% risk increase in prescriptions collection during August 2014. For August 2013--July 2014, the conditional frailty model results are in favour of the intervention group (2.9% increased risk of prescription collection), however are statistically non-significant (IR: 1.025, 95% CI: 0.971, 1.082, $p$=0.369).

**Table G.3**: Conditional frailty model results for group allocation (prescriptions).

| Time Period | Intensity Ratio (IR) | 95% Confidence Interval | p-value |
| --- | --- | --- | --- |
| Aug-13 | 1.331 | (1.167, 2.259) | <0.001 |
| Aug-14 | 1.029 | (0.875, 1.209) | 0.732 |
| Aug-13 – Jul-14 | 1.025 | (0.971, 1.082) | 0.369 |

**Table G.4**: Conditional frailty model results for group allocation (prescriptions, with rare event bias adjustment), for time periods Aug 2013 and Aug 2014.

| Time Period | Intensity Ratio (IR) | 95% Confidence Interval | p-value |
| --- | --- | --- | --- |
| Aug-13 | 1.295 | (1.176, 1.427) | <0.001 |
| Aug-14 | 1.018 | (0.909, 1.141) | 0.752 |

# H Prescriptions Sensitivity Analysis

## H.1 Truncation Points

Table H.1 shows the maximum number of prescriptions per participant for each truncated dataset (includes event risk sets above; 5%, 2%, 1%, 0.5%, 0.25% of the population), for each time period. Events per participant are capped at 1-2 events for August 2013 and August 2014, but 8-14 events for August 2013--July 2014. Without truncation, maximum number of events per participant are 3, 3, 22 for August 2013, August 2014 and August 2013--July 2014, respectively.

**Table H.1**: Maximum number of prescriptions per participant by time period and truncated dataset (includes event risk sets above the population percentage).

| Time Period | No truncation | >5% | >2% | >1% | >0.5% | >0.25% |
| --- | --- | --- | --- | --- | --- | --- |
| Aug-13 | 3 | 1 | 1 | 1 | 2 | 2 |
| Aug-14 | 3 | 1 | 1 | 1 | 2 | 2 |
| Aug-13 – Jul-14 | 22 | 8 | 11 | 12 | 13 | 14 |

Event dependence is evident in cumulative hazard plots for these truncated datasets, as well as clustering using the likelihood ratio test. Hence, the model diagnostics indicate that the conditional frailty model is appropriate for these data (results not given as these are very similar to the full event risk sets).

## H.2 Truncated Conditional Frailty Results

Table H.2 gives results of the conditional frailty model using the truncated datasets for prescriptions, for August 2013 and August 2014. Truncation points are grouped into columns, as the event cap is the same for >5% and >1%, which is 1 event, as well as for >0.5% and >0.25%, which is 2 events. Clearly, capping the number of prescriptions to a maximum of 1 per participant, means the analysis is no longer a recurrent events analysis, reverting to a time to first event analysis. A rare events bias adjustment is not applied for the truncated datasets capped at 1 event, as Table G.1 shows reasonable counts of participants achieving 1 event (August 2013: 1483 participants (12.8% of the primary population), August 2014: 1059 participants (9.2% of the primary population)).

For August 2013, analysing a 1 event maximum, the intensity ratio (IR) is 0.035 larger (same direction) than the full risk sets results, with a 0.743 narrower confidence interval (IR: 1.366, 95% CI: 1.202, 1.551), showing higher precision. For 2 events, the intensity ratio is similar to the full results, but higher precision (0.740 narrower confidence interval). Applying a rare events adjustment further reduces confidence interval width by 0.100, which is consistent with the full risk sets results with rare events adjustment (IR: 1.296, 95% CI: 1.177, 1.429). All August 2013 results are statistically significant, with strong evidence ($p<0.001$) that the intervention group experience increased risk of a prescription (uptake) at any particular time point, compared to the control group.

Results for August 2014 remain statistically non-significant, with no evidence to suggest a group difference. When capping events to 1 event, the intensity ratio is increased to 1.075 (same direction) with a 0.028 wider confidence interval. Whereas, capped at 2 events, the intensity ratio is reduced to 1.027 (same direction). Using a rare events adjustment for the 2 events capped dataset, produces results consistent with the full risk sets dataset with adjustment (IR: 1.018, 95% CI: 0.909, 1.140, $p$=0.761), in terms of effect size, direction, uncertainty (confidence interval width) and statistical significance.

**Table H.2**: Conditional frailty model results for group allocation (prescriptions), for the truncated datasets, for time periods Aug 2013 and Aug 2014.

|  |  |  | plus Firth's Adjustment |
| --- | --- | --- | --- |
| Time Period | >5% & >1% | >0.5% & >0.25% | >0.5% & >0.25% |
| Aug-13 |  |  |  |
| IR | 1.366 | 1.334 | 1.296 |
| 95% CI | (1.202, 1.551) | (1.170, 1.522) | (1.177, 1.429) |
| p-value | p < 0.001 | p < 0.001 | p < 0.001 |
| Aug-14 |  |  |  |
| IR | 1.075 | 1.027 | 1.018 |
| 95% CI | (0.909, 1.271) | (0.873, 1.209) | (0.909, 1.140) |
| p-value | p = 0.399 | p = 0.748 | p = 0.761 |

Results for August 2013--July 2014 using the conditional frailty model and truncated datasets are given in Table H.3. These are consistent to using the full risk sets, in terms of effect size, direction, uncertainty and statistical significance.

**Table H.3**: Conditional frailty model results for group allocation (prescriptions), for the truncated datasets, for Aug 2013 – Jul 2014.

| Time Period | >5% | >2% | >1% | >0.5% | >0.25% |  |
| --- | --- | --- | --- | --- | --- | --- |
| Aug-13 - Jul-14 | |  |  |  |  |  |
| IR | 1.033 | 1.03 | 1.028 | 1.027 | 1.027 |  |
| 95% CI | (0.978,1.090) | (0.976,1.087) | (0.974,1.085) | (0.974,1.084) | (0.972,1.085) | |
| p-value | p = 0.250 | p = 0.287 | p = 0.313 | p = 0.326 | p = 0.350 |  |

# References

**29 Balan TA and Putter H.** A tutorial on frailty models. Stat Methods Med Res 2020; 29: 3424–3454.

**38 Therneau TM and Grambsch PM.** Modeling survival data: extending the Cox model. New York: Springer, 2000.

**39 Therneau TM.** A package for survival analysis in R. R package version 3.2-11, <https://CRAN.R-project.org/package=survival>, 2021b.

**40 Heinze G, Ploner M and Jiricka L**. coxphf: Cox regression with Firth's penalized likelihood. R package version 1.13.1, <https://CRAN.R-project.org/package=coxphf>, 2020.

**42 Abreu AM and Sousa-Ferreira I**. A review of Cox’s model extensions for multiple events. IJRDO J Appl Sci 2019; 5: 47–62.

**51 Pedersen SE, Hurd SS, Lemanske Jr RF, et al.** Global strategy for the diagnosis and management of asthma in children 5 years and younger. Paediatr Pulmonol 2011; 46: 1–17.

**52 Menard SW**. Logistic Regression. London: Sage, 2010.

**53 Bates D, Maechler M, Bolker B, et al.** Fitting linear mixed-effects models using lme4. J Stat Softw 2015; 67: 1–48.

**54 Lele SR, Keim JL and Solymos P**. ResourceSelection: resource selection (probability) functions for use-availability data. R package version 0.3-5, <https://CRAN.R-project.org/package=ResourceSelection>, 2019.

**55 Hosmer DW**. Applied logistic regression. 3rd ed. Chichester: Wiley, 2013.

**56 Hilbe JM**. Negative binomial regression. 2nd ed. Cambridge: Cambridge University Press, 2011.

**57 Therneau TM**. coxme: mixed effects Cox models. R package version 2.2-16, <https://CRAN.R-project.org/package=coxme>, 2020.

**58 Kassambara A, Kosinski M and Biecek P**. survminer: drawing survival curves using ‘ggplot2’. R package version 0.4.9. <https://CRAN.R-project.org/package=survminer>, 2021.

**59 R Core Team**. R: A language and environment for statistical computing (2021), R Foundation for Statistical Computing, Vienna, Austria, <https://www.R-project.org/>
